# Human zygotic genome activation is initiated from paternal genome

**DOI:** 10.1038/s41421-022-00494-z

**Published:** 2023-01-31

**Authors:** Shenli Yuan, Jianhong Zhan, Jingye Zhang, Zhenbo Liu, Zhenzhen Hou, Chuanxin Zhang, Lizhi Yi, Lei Gao, Han Zhao, Zi-Jiang Chen, Jiang Liu, Keliang Wu

**Affiliations:** 1 Center for Reproductive Medicine, Shandong University, Jinan, Shandong China; 2 CAS Key Laboratory of Genome Sciences and Information, Collaborative Innovation Center of Genetics and Development, Beijing Institute of Genomics, and China National Center for Bioinformation, Chinese Academy of Sciences, Beijing, China; 3 University of Chinese Academy of Sciences, Beijing, China; 4 Key laboratory of Reproductive Endocrinology of Ministry of Education, Shandong University, Jinan, Shandong China; 5 Center for Reproductive Medicine, Ren Ji Hospital, School of Medicine, Shanghai Jiao Tong University, Shanghai, China; 6 CAS Center for Excellence in Animal Evolution and Genetics, Chinese Academy of Sciences, Kunming, Yunnan China

**Keywords:** Reprogramming, Transcriptomics

## Abstract

Although parental genomes undergo extensive epigenetic reprogramming to be equalized after fertilization, whether they play different roles in human zygotic genome activation (ZGA) remains unknown. Here, we mapped parental transcriptomes by using human parthenogenetic (PG) and androgenetic (AG) embryos during ZGA. Our data show that human ZGA is launched at the 8-cell stage in AG and bi-parental embryos, but at the morula stage in PG embryos. In contrast, mouse ZGA occurs at the same stage in PG and AG embryos. Mechanistically, primate-specific ZNF675 with AG-specific expression plays a role in human ZGA initiated from paternal genome at the 8-cell stage. AG-specifically expressed LSM1 is also critical for human maternal RNA degradation (MRD) and ZGA. The allelic expressions of ZNF675 and LSM1 are associated with their allelically epigenetic states. Notably, the paternally specific expressions of ZNF675 and LSM1 are also observed in diploid embryos. Collectively, human ZGA is initiated from paternal genome.

## Introduction

After fertilization, mammalian embryo undergoes maternal to zygotic transition (MZT), in which maternally deposited RNAs are degraded while zygotic genome begins to be transcribed. Maternal RNA degradation (MRD) and zygotic genome activation (ZGA) are two highly correlated events^1–3^, which are essential for ensuing the first lineage decisions^4^. Although the MZT is conserved across the kingdom animalia, the timing of ZGA is remarkably different. Human ZGA mainly occurs at the 8-cell stage, while mouse ZGA occurs at the late 2-cell stage. The mechanisms regulating ZGA are extensively investigated. It is well-established that the timely release of maternal activators and removal of maternal repressors ensure the right timing of ZGA^5,6^. Recent studies have shown that human ZGA is associated with the dynamics of epigenetic states of chromatin^7,8^. Several maternal activators that directly bind to ZGA genes and alter the chromatin states have been identified in many organisms^5^. For example, OCT4 is important for the chromatin accessibility of ZGA genes in human embryos^7^, and NFYA regulates the chromatin accessibility of ZGA genes in mouse embryos^9^. DUX4 plays an important role in human ZGA, as the binding motif of DUX4 is enriched at the promoter regions of the human ZGA genes^10^. In addition, many transposons including ERVL and LINE1 are activated in the cleavage embryos, which has been confirmed to be indispensable for activation of many ZGA genes^11–13^. On the contrary, it has also been reported that, in Piwil1-deficient embryos, abnormal activation or persistent existence of endogenous retroviruses (ERVs) was associated with impaired ZGA and the decay of maternally deposited transcripts^14^. Although the epigenetic dynamics and transcription activators have been investigated during human MZT^7,8,15–18^, it is still poorly understood how MZT is switched on during human embryogenesis.

As we know, the epigenetic states between sperm and oocyte are extraordinarily different. After fertilization, although both parental genomes go through extensive reprogramming to be epigenetically equalized, a portion of genomic regions are still epigenetically distinct between paternal and maternal genomes, which are associated with allelic-specific gene expression in early embryos^19,20^. The properly allelic-specific epigenetic states of chromatin are essential for normal development. For example, genome imprinting is usually regulated by allelic DNA methylation. Abnormal genome imprinting leads to growth retardation and several kinds of human disorders, such as Angelman Syndrome (AS)^21^. It is well established that mammalian parthenogenetic (PG) and androgenetic (AG) embryos cannot develop to term due to imprinting defects. AG embryos can form blastocysts, but cannot pass early somite stages, which usually contain abundant extraembryonic tissues. In contrast, PG embryos can occasionally develop to later somite stages, but lack extraembryonic tissues^21^. It suggests that maternal and paternal genomes play unequal roles in regulating embryonic development. Using single nucleotide polymorphisms (SNPs) to distinguish paternal and maternal genomes has been widely used in many inbred species including mouse^20,22–24^. However, due to the mixed genetic background, informative SNPs are only detected in a limited proportion of human genomes^25^. As a result, our knowledge about the patterns and roles of parental genomes is limited during human development. In particular, it is interesting to know whether paternal and maternal genomes play different roles during the processes of MRD and ZGA in human.

Previous studies have indicated that PG and AG genomes can mimic maternal and paternal genomes during mouse early embryogenesis, respectively^26–29^. Human PG and AG embryos have been established in the last decade, which are useful models to investigate the differences between maternal and paternal genomes^30,31^. Recently, Leng et al. used human PG and AG embryos, and found that gene expression patterns between PG and AG embryos during ZGA are different^32^. They found that maternally biased expressed genes (MBGs) became apparent at the 4-cell stage and contributed to the initiation of ZGA, whereas paternally biased expressed genes (PBGs) preferentially appeared at the 8-cell stage and might affect embryo compaction and trophectoderm specification. They also demonstrated that the parentally specific DNA methylation might account for the expression of most PBGs. This study indicates that the gene expression patterns and epigenetic states between parental genomes are distinct during MZT, which implies that the paternal genomes may exert different roles in ZGA. However, they did not clarify whether the gene expression differences between PG and AG embryos are arisen from MRD or ZGA. More importantly, the underlying molecular mechanisms regulating the differential gene expression between human PG and AG embryos remain unknown.

## Results

### Human ZGA is activated in AG embryos but delayed in PG embryos at the 8-cell stage

**Fig. 1:**
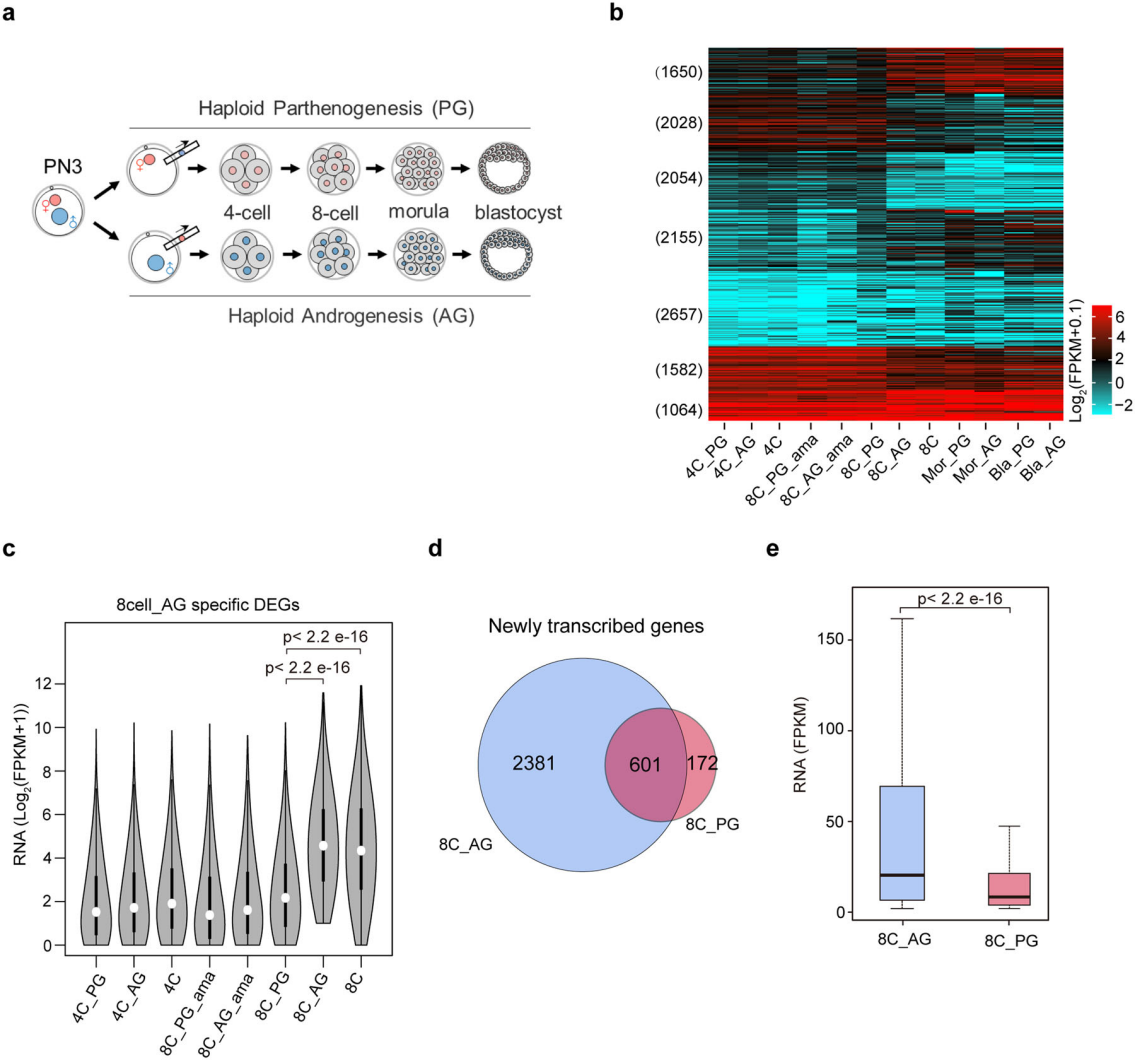
Human ZGA is activated in AG embryos but delayed in PG embryos at the 8-cell stage. **a** Schematic of the generation of human haploid parthenogenetic (PG) and androgenetic (AG) embryos at early stages, which were used for RNA-seq. **b** Heatmap showing the expression levels of genes in human haploid embryos, α-amanitin (ama) treated haploid embryos and bi-parental embryos. The genes expressed at least one sample are clustered by k-means method. The gene number in each cluster is shown on the left. 4 C represents 4-cell embryos; 8 C represents 8-cell embryos; Mor represents morula; Bla represents blastocyst. **c** Violin plots comparing the expression levels of differential expressed genes (DEGs) with AG-specific expression at the 8-cell stage in the indicated human embryos. Wilcoxon rank sum test was used. **d** Venn diagram comparing the newly transcribed genes between PG and AG 8-cell embryos. The genes, whose expression levels are significantly higher at 8-cell stage comparing to 4-cell stage, are defined as newly transcribed genes. **e** Box plot comparing the expression levels of newly transcribed genes between AG and PG 8-cell embryos. Wilcoxon rank sum test was used.

To explore the contributions of parental genomes to human ZGA, we generated and collected PG and AG embryos at the 4-cell, 8-cell, morula and blastocyst stages (see “Materials and methods”) (Fig. 1a). The embryos with high morphological qualities were collected to map the transcriptomes (Fig. 1a and Supplementary Table S1). We firstly compared the gene expression patterns among PG, AG and bi-parental (also called diploid) embryos during MZT. Our data show that transcript profiles between PG and AG embryos at the 4-cell stage are similar, both of which resemble that in bi-parental 4-cell embryos (Fig. 1b and Supplementary Fig. S1a). However, the transcriptomes between PG and AG embryos at the 8-cell stage are extremely distinct. The transcriptome of AG embryos at the 8-cell stage is similar to that of bi-parental embryos at the 8-cell stage, whereas the transcriptome of PG embryos at the 8-cell stage is similar to that of bi-parental 4-cell embryos rather than 8-cell embryos (Fig. 1b and Supplementary Fig. S1a). It implies that ZGA is extensively activated in AG embryos but not PG embryos at the 8-cell stage. To support our speculation, we compared the transcriptomes between PG and AG embryos at the 8-cell stage. There are 1881 differentially expressed genes (DEGs) with AG-specifically high expression and 1403 DEGs with PG-specifically high expression (Supplementary Fig. S1b). The AG-specific DEGs at the 8-cell stage are enriched in the RNA metabolism and translation while the PG-specific DEGs are enriched in the tissue morphogenesis and development (Supplementary Table S2). For the 1881 genes with AG-specific expression, most of them show higher expression levels in AG 8-cell embryos than both bi-parental and AG 4-cell embryos (Fig. 1c). It suggests that the AG-specific genes in 8-cell embryos should be nascently transcribed at the 8-cell stage. To validate this hypothesis, we compared the transcriptomes of AG embryos between 4-cell and 8-cell stages, and identified 2982 nascently transcribed genes (also can be called as the ZGA genes) in 8-cell AG embryos. 87.2% (1640 in 1881) of AG-specifically expressed genes at the 8-cell stage are ZGA genes (Supplementary Fig. S1c), supporting that these AG-specific transcripts at the 8-cell stage mainly come from ZGA. Our data also show that although 773 genes are newly transcribed in PG 8-cell embryos (Fig. 1d), the overall expression levels of these 773 genes in PG 8-cell embryos are much lower than those of 2982 ZGA genes in AG 8-cell embryos (Fig. 1e). We also noticed that 3509 genes showed significantly higher expression levels in PG morulae than PG 4-cell embryos, and 2202 of the 3509 genes are ZGA genes in AG 8-cell embryos (Supplementary Fig. S1d), demonstrating that ZGA is just delayed in PG embryos at the 8-cell stage. Moreover, we analyzed the DEGs between PG and AG embryos at the morula and blastocyst stages (Supplementary Fig. S1b). At the morula stage, the AG-specific DEGs are enriched in the negative regulation of myoblast differentiation, while the PG-specific DEGs are enriched in the detoxification. At the blastocyst stage, the AG specific DEGs are enriched in the carbohydrate metabolic process and ncRNA metabolic process, while the PG specific DEGs are enriched in the glial cell apoptotic process (Supplementary Table S2).

To further confirm that ZGA is activated in AG embryos but delayed in PG embryos at the 8-cell stage, we treated embryos with α-amanitin, which is a specific inhibitor of RNA polymerase II, to block ZGA^15^. As expected, upon α-amanitin treatment, the expression of ZGA genes is repressed in AG 8-cell embryos (Supplementary Fig. S1e). Besides, the transcriptome of α-amanitin treated AG 8-cell embryos is similar to that of AG and bi-parental 4-cell embryos (Fig. 1b, c). Our results also show that the transcriptome of 8-cell PG embryos is similar to those of α-amanitin treated PG (8C_PG_ama) or AG embryos (8C_AG_ama) at the 8-cell stage (Supplementary Fig. S2a), supporting that ZGA is not fully activated in PG embryos at the 8-cell stage. In addition, to explore whether the delay of ZGA in PG embryos at the 8-cell stage is caused by the growth retardation, we checked the developmental time points of human PG and AG haploid embryos as well as diploid embryos from the 2-cell stage to 8-cell stage after fertilization. The developmental time points of human embryos at 2-cell, 4-cell or 8-cell stages were comparable between PG and AG embryos (Supplementary Fig. S2b). This result suggests that the paternally biased expression is not caused by the growth retardation.

Taken together, human ZGA is activated in AG embryos but delayed in PG embryos at the 8-cell stage, suggesting that human ZGA is initiated from the paternal genome.

### Mouse ZGA is activated in both AG and PG embryos at the late 2-cell stage

Next, we expect to know whether the delayed ZGA of maternal genome could also be detected in mouse embryos. We collected mouse late 2-cell AG and PG embryos by utilizing a similar method with human haploid embryo (see Materials and methods). By comparing the transcriptomes of the late 2-cell AG and PG embryos, we only found 421 differentially expressed genes (DEGs) with AG-specifically high expression and 298 DEGs with PG-specifically high expression (Supplementary Fig. S3a). The numbers of DEGs between AG and PG embryos at the ZGA stage in mouse are extremally lower than those in human. Then, we were curious about how many ZGA genes were differentially expressed between AG and PG embryos at the late 2-cell stage in mouse. Our data show that there are only 63 ZGA genes with AG-specifically high expression and 100 ZGA genes with PG-specifically high expression (Supplementary Fig. S3a). It suggests that the ZGA is not delayed in mouse late 2-cell PG embryos. Consistently, the expression levels of ZGA genes are comparable between mouse AG and PG embryos (Supplementary Fig. S3b). Furthermore, clustering analysis shows that the transcriptomes of both late 2-cell AG and PG embryos are similar to that of late 2-cell bi-parental embryos rather than that of α-amanitin treated late 2-cell embryos (Supplementary Fig. S3c)^20^. It indicates that ZGA is activated both in late 2-cell AG and PG embryos in mouse.

To further confirm this conclusion, we investigated the allelic expression of the ZGA genes in mouse bi-parental embryos at the late 2-cell stage, in which the expression of parental alleles can be tracked by the SNPs between C57BL/6N and PWK/PhJ mouse strains^33^. There are only 56 ZGA genes with paternally high expression and 173 ZGA genes with maternally high expression (Supplementary Fig. S3d). It supports that ZGA in maternal genome is not delayed in mouse embryos at the late 2-cell stage.

Taken together, the initiation of ZGA firstly from paternal genome is only observed in human early embryos, which is not conserved between human and mouse.

### Paternally activated ZNF675 promotes ZGA

**Fig. 2:**
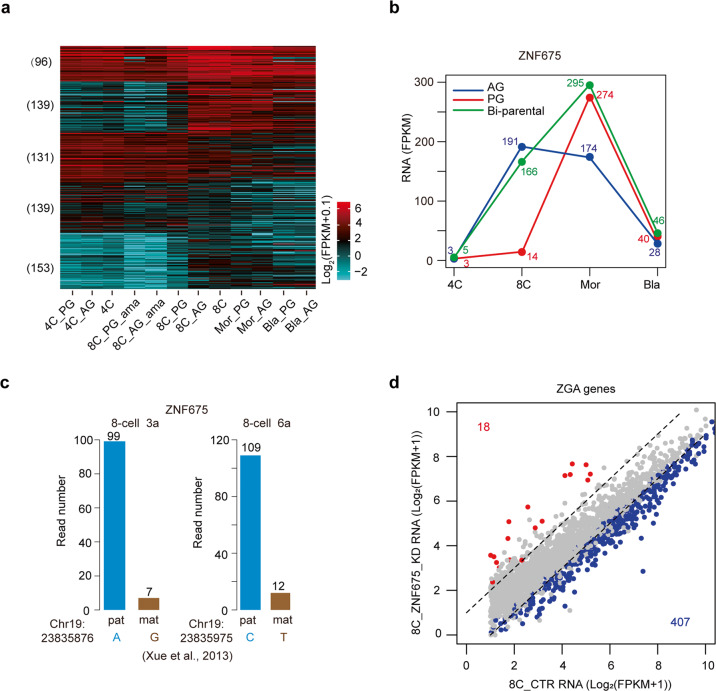
Paternally expressed ZNF675 contributes to ZGA. **a** Heatmap showing the expression levels of transcription factors (TFs) in human haploid embryos, α-amanitin (ama) treated haploid embryos and bi-parental embryos. The TFs expressed at diploid 8-cell embryos are clustered by k-means method. **b** Plot showing the expression levels of ZNF675 in human haploid and bi-parental embryos. **c** Bar plot showing the read numbers for ZNF675 transcripts from paternal and maternal genomes in human bi-parental 8-cell embryos (samples 3a and 6a). The RNA-seq data are from^35^. The locations of SNPs, and the alleles at these SNP loci in maternal (mat) and paternal (pat) genomes are indicated. **d** Scatter plot showing the expression levels of ZGA genes between ZNF675 KD and control (CTR) 8-cell embryos. The genes upregulated in ZNF675 KD embryos are labeled in red, while the genes downregulated in ZNF675 KD embryos are labeled in blue. The numbers of ZGA genes belonging to DEGs are indicated.

Transcription factors (TFs) play key roles in ZGA^7,9^. To find out the mechanism about how ZGA is activated at the 8-cell stage in human AG and bi-parental embryos but not in PG embryos, we hypothesized that some TFs specifically expressed in paternal genome were required for ZGA. To prove it, we firstly investigated the expression patterns of TFs, downloaded from (http://humantfs.ccbr.utoronto.ca), in human haploid and bi-parental embryos. Our data show that the expression patterns of the TFs in PG 8-cell embryos are similar to those in the embryos at the 4-cell stage, while the expression patterns of the TFs in AG 8-cell embryos are similar to those in bi-parental 8-cell embryos (Fig. 2a). Furthermore, our data show that there are 179 TFs with AG-specific expression, and 118 TFs with PG-specific expression at the 8-cell stage (Supplementary Fig. S4a). Next, because the ZGA delay in PG embryos at the 8-cell stage is only observed in human but not in mouse, we speculated that some primate- or human-specific transcription factors (TFs) specifically expressed in AG embryos are responsible for the ZGA in human. To find out such TFs, we focused on the TFs with AG-specific expression at the 8-cell stage. Besides, the candidate TFs should also show high expression levels in biparental human 8-cell embryos and human PG morulae. We further ranked the candidate TFs according to the expression differences between AG and PG 8-cell embryos in descending order. Among the top five TFs, ZNF675 is the only one gene that is primate-specific (Supplementary Table S3). It is specifically expressed in AG 8-cell embryos with the striking difference of expression levels between AG and PG embryos (Fig. 2b). In addition, ZNF675 is highly expressed in bi-parental 8-cell embryos (Fig. 2b). Although ZNF675 shows relatively low expression level in PG 8-cell embryos, its expression level in PG morulae is high (Fig. 2b). Moreover, we also checked the expression of ZNF675 in human early embryos by using the published single-cell RNA-seq data^34^. The expression of ZNF675 can be detected in human oocyte and early embryos at the zygote, 2-cell and 4-cell stages before major ZGA (Supplementary Fig. S4b). The expression level of ZNF675 is largely elevated at the 8-cell stage (Supplementary Fig. S4b). To further confirm that ZNF675 is expressed in human early embryos before ZGA stage, based on the observation that ZNF675 protein can be detected in HEK 293T cells (Supplementary Fig. S4c), we compared the expression levels of ZNF675 between human early embryos and HEK 293T cells by using quantitative PCR. Our data show that the mRNA levels of ZNF675 in human early embryos from 2-cell stage to 8-cell stage are at least eight-fold higher than that in HEK 293T cells (Supplementary Fig. S4d). The expression level of ZNF675 in human 4-cell embryos is about a half of that in human 8-cell embryo (Supplementary Fig. S4d). These results support that ZNF675 is expressed in human early embryos before ZGA stage. Thus, ZNF675 mRNA is maternal deposited and actively transcribed at ZGA stage. To confirm the paternally specific expression of ZNF675 in human 8-cell embryos, we analyzed previously published single-cell RNA-seq data of human early embryos, in which the gene expression levels between paternal and maternal genomes could be distinguished by SNPs^35^. Several SNPs can be identified in the exons of ZNF675. Consistently, ZNF675 shows paternally specific expression in the 8-cell blastomeres (Fig. 2c) (see “Materials and methods”). Collectively, ZNF675 is specifically expressed in paternal genome rather than maternal genome at the ZGA stage.

To answer whether the paternally specific expression of ZNF675 is essential for ZGA, we knocked down ZNF675 in human bi-parental 8-cell embryos. The expression of ZNF675 at the 8-cell stage is largely diminished upon ZNF675 knockdown (KD) (Supplementary Fig. S4e). Among the three different ZNF675 siRNAs, ZNF675 siRNA #1 exerts a better KD effect than siRNA #2 and siRNA #3 (Supplementary Fig. S4e). To exclude the off-target effects of ZNF675 siRNAs, we assessed the gene expression in ZNF675 KD embryos in which ZNF675 was knocked down by ZNF675 siRNA #1 and #3, respectively. Our data show that 1005 genes downregulated in ZNF675 KD embryos and 593 genes upregulated in ZNF675 KD embryos (Supplementary Fig. S5a). The genes downregulated in ZNF675 KD embryos are enriched in the RNA metabolism while the genes upregulated in ZNF675 KD embryos are enriched in the development and cell morphogenesis (Supplementary Fig. S5b). In the ZNF675 KD embryos, 19.4% (407/2095) of ZGA genes are significantly downregulated (Fig. 2d). Previous works have reported that OCT4 and DUX4 are important for human ZGA^7,10^. To further evaluate the regulatory functions among ZNF675, OCT4, and DUX4 in human ZGA, we compared the downregulated ZGA genes among ZNF675 KD, OCT4 KD^7^ and DUX4 KD^10^ human 8-cell embryos. There are 210 ZGA genes that are downregulated both in ZNF675 KD and OCT4 KD embryos (Supplementary Fig. S5c). However, only 20 ZGA genes are downregulated in DUX4 KD embryos, which is consistent with the fact that DUX4 is lowly expressed in human 8-cell embryos, and DUX4 KD leads to minor changes in the embryonic transcriptome^10^. Among them, 5 ZGA genes are downregulated both in ZNF675 KD and DUX4 KD embryos. Taken together, paternally activated ZNF675 is important for the ZGA. It is interesting that ZNF675 is a new gene which can be found only in primate. This may be one reason that the mechanism of ZGA in human is different from that in mouse.

Previous works have proven that ZGA is essential for the maternal mRNA degradation^5^. To answer whether the paternally activated ZNF675 is also essential for maternal RNA degradation, we investigated the expression levels of the maternally degraded genes in ZNF675 KD embryos at the 8-cell stage. Our data show that 438 maternally degraded genes are upregulated in ZNF675 KD embryos (Supplementary Fig. S5d). This suggests that ZNF675 KD results in the degradation failure of the maternal mRNAs, which may be caused by the defects in ZGA upon ZNF675 KD.

### Paternally activated MRD is essential for ZGA

**Fig. 3:**
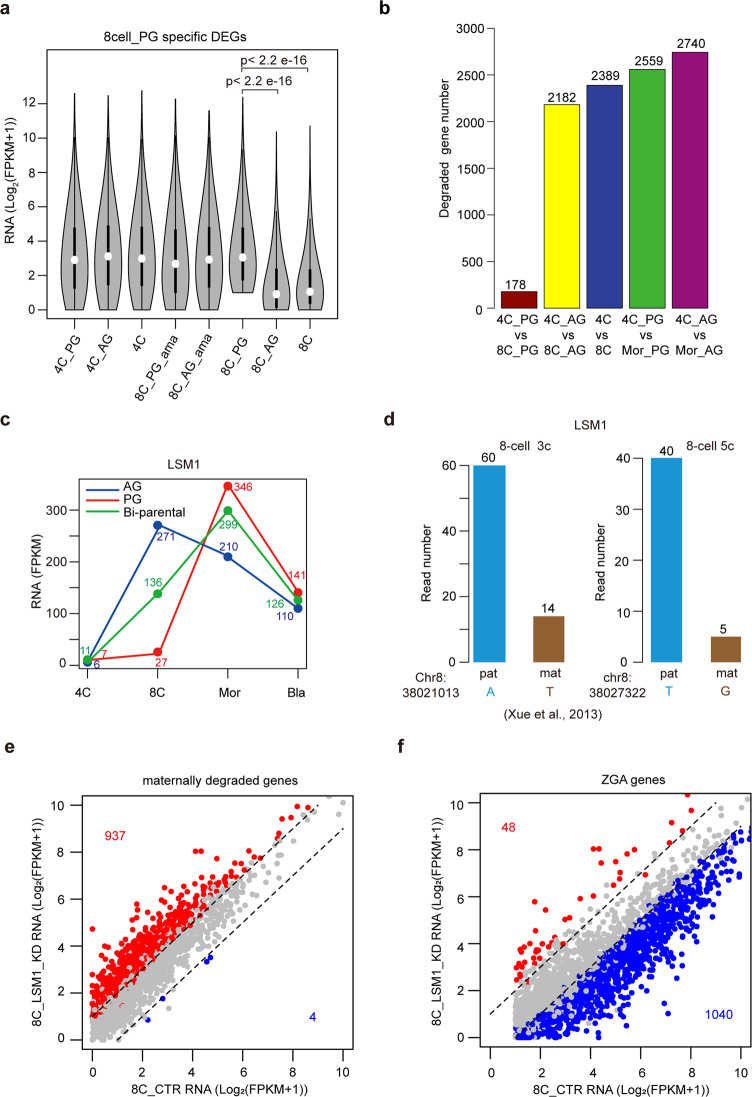
Paternally activated MRD is essential for ZGA in human. **a** Violin plots showing the expression levels of PG specifically expressed genes at the 8-cell stage in the indicated human embryos. Wilcoxon rank sum test was used. **b** Bar plot showing the numbers of maternal genes whose transcripts are degraded in haploid or bi-parental embryos from 4-cell to 8-cell or morula stages. **c** Plot showing the expression levels of LSM1 in human haploid and bi-parental embryos. **d** Bar plot showing the read numbers for LSM1 transcripts from paternal and maternal genomes in human bi-parental 8-cell embryos (samples 3c and 5c). The RNA-seq data are from^35^. The locations of SNPs, and the alleles at these SNP loci in maternal and paternal genomes are indicated. **e** Scatter plot showing the expression levels of maternally degraded genes between LSM1 KD and control 8-cell embryos. The genes upregulated in LSM1 KD embryos are labeled in red, while the genes downregulated in LSM1 KD embryos are labeled in blue. The numbers of maternally degraded genes belonging to DEGs are indicated. **f** Scatter plot showing the expression levels of ZGA genes between LSM1 KD and control 8-cell embryos. The genes upregulated in LSM1 KD embryos are labeled in red, while the genes downregulated in LSM1 KD embryos are labeled in blue. The numbers of ZGA genes belonging to DEGs are indicated.

It is well-known that MRD and ZGA are two concomitant events. It is reported that the activation of MRD is critical for ZGA^5^. We were curious about whether the MRD pathway was also responsible for the initiation of human ZGA from paternal genome. Thus, we examined the MRD in human 8-cell PG and AG embryos. We firstly assessed the expression levels of the 1403 PG-specific genes (PG vs AG at the 8-cell stage) among different human early embryos. Our data show that the expression levels of most of these genes in PG 8-cell embryo are comparable to those in AG, PG or bi-parental 4-cell embryos, but are higher than those in bi-parental or AG 8-cell embryos (Fig. 3a). It suggests that the PG-specific genes (PG vs AG) at the 8-cell stage may be maternally expressed genes. The accumulation of them in PG 8-cell embryos may be caused by the failure of maternal mRNA degradation. To confirm the defect of maternal mRNA degradation in PG 8-cell embryo, we compared the transcriptomes of bi-parental embryos between 4-cell and 8-cell stages to identify the degraded maternal genes. 2389 genes are degraded in bi-parental embryo at the 8-cell stage (Fig. 3b). Comparably, 2182 genes are degraded in AG embryos from 4-cell stage to 8-cell stage (Fig. 3b). In contrast, only 178 genes are degraded in PG embryos from 4-cell stage to 8-cell stage (Fig. 3b). Our data also show that 70.8% (994/1403) of PG-specific genes are overlapped with the degraded genes in AG embryos at the 8-cell stage (Supplementary Fig. S6a). These results support that maternal RNA degradation is only activated in AG embryos but paused in PG embryos at the 8-cell stage. In addition, we noticed that most of maternally deposited RNA were degraded in PG morula (Figs. 1b, 3b). Taken together, our data indicate that the MRD is activated by paternal genome rather than maternal genome at the 8-cell stage, which may facilitate the ZGA in human embryo. In contrast, the delay of MRD in PG embryo at the 8-cell stage is associated with the ZGA delay.

To unveil the underlying mechanisms of MRD activation in AG 8-cell embryos but not PG 8-cell embryos, we hypothesized that some factors responsible for RNA degradation were specifically transcribed from paternal genome at the 8-cell stage. To validate this hypothesis, we investigated the gene expression of the key factors participating in RNA degradation^1,36,37^ (Supplementary Fig. S6b). We noticed that the expression levels of some MRD related genes, whose expression levels are largely elevated at the 8-cell stage, are higher in AG than PG 8-cell embryos (Supplementary Fig. S6b). For example, LSM1 is specifically expressed in AG embryos but not in PG embryos at the 8-cell stage (Fig. 3c and Supplementary Fig. S6b). The paternally specific expression of LSM1 is confirmed in bi-parental 8-cell embryos, in which the gene expression levels between paternal and maternal genomes could be distinguished by SNPs^35^ (Fig. 3d). To further demonstrate whether the low expression of LSM1 in maternal genome contributes to MRD delay in PG 8-cell embryos, we knocked down LSM1 in bi-parental 8-cell embryos (Supplementary Fig. S6c). we detect 2221 genes downregulated in LSM1 knockdown (KD) embryos and 1259 genes upregulated in LSM1 KD embryos (Supplementary Fig. S7a). The genes downregulated in LSM1 KD embryos are enriched in the ribonucleoprotein complex biogenesis while the genes upregulated in LSM1 KD embryos are enriched in the development (Supplementary Fig. S7b). Our data show that LSM1 knockdown (KD) results in the degradation failure of around 40% (937/2389) of maternal mRNAs (Fig. 3e). Consistently, transcriptome profile of LSM1 KD embryos is similar to that of PG embryos at the 8-cell stage (Supplementary Fig. S7c). Although EXOSC5 also plays important roles in RNA degradation^37^, KD of EXOSC5 which is specifically expressed in AG 8-cell embryos does not affect maternal transcript degradation (Supplementary Figs. S6c, S7d). Collectively, these results indicate that LSM1-dependent RNA degradation specifically activated from paternal genome is critical for MRD in human early embryos.

To further demonstrate whether the activation of LSM1 dependent MRD is important for the ZGA in human 8-cell embryos, we examined the expression of ZGA genes in the LSM1 KD embryos. Around 50% (1040/2095) of ZGA genes are significantly downregulated in the LSM1 KD 8-cell embryos (Fig. 3f). It suggests that LSM1 associated MRD is critical for ZGA. Previous study has shown that OCT4 plays important role in human ZGA^7^. Interestingly, we observe that LSM1 is downregulated in OCT4 KD 8-cell embryos (Supplementary Fig. S7e), suggesting that the expression of LSM1 is regulated by OCT4 at the 8-cell stage.

Taken together, paternally specific expression of the transcription factor ZNF675 and paternally specific activation of LSM1-dependent MRD is important for human ZGA in the 8-cell embryos.

### Allelically epigenetic reprogramming is associated with the initiation of human ZGA from paternal genome

**Fig. 4:**
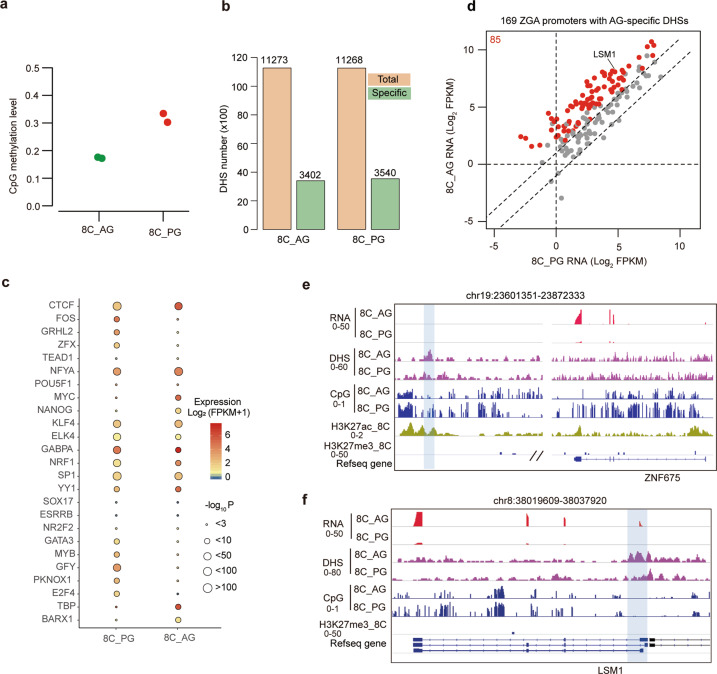
Epigenetic states at ZNF675 and LSM1 loci. **a** Plot showing the global DNA methylation levels of parental genomes in human PG and AG embryos at the 8-cell stage. **b** Bar plots showing the number of total DHSs and parentally specific DHSs in human PG and AG embryos at the 8-cell stage. **c** Transcription factor binding motif enrichment in the parentally specific DHSs at the 8-cell stage. The sizes of circles represent the p values of enrichment. The colors in the circles represent the expression levels of transcription factors in the corresponding PG or AG embryos. **d** Scatter plot showing the RNA expression of the ZGA genes whose promoters harbor AG-specific DHSs. **e** Genome browser view of RNA expression and epigenetic states at ZNF675 locus. The blue shadow indicates a putative enhancer region associated with ZNF675. **f** Genome browser view of RNA expression and epigenetic states at LSM1 locus. The blue shadow indicates the promoter region of LSM1.

Epigenetic modifications play important roles in gene expression regulation^38^. The epigenetic states of sperm and oocyte are dramatically different. After fertilization, the epigenetic modifications undergo extensive reprogramming to be epigenetically equalized. We are curious about whether the role differences of parental genomes in human ZGA are associated with allelically epigenetic states. Thus, we investigated the chromatin accessibility landscapes and DNA methylation patterns in human AG and PG embryos at the 8-cell stage. Consistent with previous reports, our data show that the genome-wide DNA methylation level of AG embryos is lower than that of PG embryo (Fig. 4a), suggesting that the paternal genome is in a more permissive state than maternal genome. Moreover, we find most of differentially methylated regions (DMRs) with PG hypermethylation are located in genic regions, while most of DMRs with AG hypermethylation are located in intergenic regions (Supplementary Fig. S8a). The genes with AG hypermethylated DMRs in the promoters are enriched in detection of chemical stimulus involved in sensory perception and activation of GTPase activity, while the genes with PG hypermethylated DMRs in the promoters are enriched in the cilium movement (Supplementary Table S4).

Next, we compared the chromatin accessibility landscapes between AG and PG embryos at the 8-cell stage. Although the DHS (DNase I hypersensitive site) numbers in AG and PG embryos are comparable, about one third of DHSs show differential DHS signals between AG and PG embryos (Fig. 4b). Moreover, we find most of the PG and AG-specific DHSs are located in promoters and intergenic regions. (Supplementary Fig. S8b). The genes with AG-specific DHSs in the promoters are enriched in the metabolism of RNA and translation, while the genes with PG-specific DHSs in the promoters are enriched in cell cycle and ncRNA metabolic process (Supplementary Table S5). Because DHSs are usually cis-elements bound by TFs to activate gene expression, we analyzed the TF binding motif enrichment in the AG and PG specific DHSs. Our data show that NANOG binding motif is enriched in the AG specific DHSs but not PG specific DHSs (Fig. 4c). Previous works have revealed that NANOG, OCT4, and SOX2 form a regulation network to regulate the expression of pluripotent genes^39^, and OCT4 and SOX2 are important for human ZGA^7^. It provides some hints that NANOG may play some roles in human ZGA initiated from paternal genome. Moreover, we also examined the DNA methylation levels and chromatin accessibility of the promoters of all ZGA-related TFs in human PG an AG 8-cell embryos^40^. As shown in Supplementary Fig. S9a, most of the ZGA-related TFs show low DNA methylation both in AG and PG 8-cell embryos. Moreover, 136 promoters of ZGA-related TFs are open both in AG and PG 8-cell embryos. 51 promoters of ZGA-related TFs are only open in AG 8-cell embryos while 47 promoters of ZGA-related TFs are only open in PG 8-cell embryos (Supplementary Fig. S9b).

To answer whether the initiation of ZGA from paternal genome is caused by the AG-specific chromatin accessibility, we then analyzed the expression of ZGA genes whose promoters harbor AG-specific DHSs (*n* = 247 genes). Our data show that about 50.3% (85/169) of ZGA genes with AG-specific DHSs show AG-specific expression (Fig. 4d). We can also detect 231 ZGA genes whose promoters harbor PG-specific DHSs (*n* = 231 genes). However, 42.9% (99/231) of the genes show AG-specific expression, and none of them show PG-specific expression because of the ZGA delay in PG 8-cell embryos (Supplementary Fig. S9c). One possible explanation for this result is that these genes with PG-specific promoter DHSs are primed to be expressed, and other allelic *cis*-elements or factors may participate in the activation of these genes in AG 8-cell embryos. We further examined the epigenetic states of *cis*-elements around ZNF675 and LSM1 in AG and PG 8-cell embryos. A putative enhancer with AG-specific DHS signal is observed in the downstream of ZNF675 gene (Fig. 4e). In addition, this enhancer is hypomethylated in AG 8-cell embryos, but hypermethylated in PG 8-cell embryos (Fig. 4f), which fits the paternal-specific expression of ZNF675. Besides, we observed that the promoter of LSM1 is unmethylated in both AG and PG embryos, but it has an AG-specific DHS (Fig. 4f). It may suggest that the establishment of open chromatin state in LSM1 promoter in paternal genome is important for the paternally specific expression of LSM1.

Taken together, the allelic specific epigenetic states are associated with paternally specific activation of ZNF675 and LSM1, which plays an important role in human ZGA.

## Discussion

It is an important question about how the zygotic genome is activated during human early embryonic development. Unexpectedly, our data show that paternal genome plays an important role during MRD and ZGA in human early embryos. This result suggests that paternal genomic landscape is very crucial for human development. It would be interesting to know whether the infertility of some patients is caused by the defect of paternal genome in activating human ZGA.

Different from human, no data show that paternal and maternal genomes play distinguished roles on MRD and ZGA in mouse. In addition, a recent study has also shown that OCT4 regulates human ZGA but not in mouse^7^. These data suggest that there are significantly different regulatory mechanisms regulating ZGA during early embryogenesis between human and mouse. Besides the ZGA process, the epigenetic reprogramming between human and mouse also present significant differences, such as high-order chromatin structure^15,20,24^ and H3K27me3 patterns^18,41,42^. In the future, more studies are needed to investigate the differences of early embryogenesis between human and mouse.

Our data show that ZGA from paternal genome takes place at the 8-cell stage, but maternal genome ZGA takes place at the morula stage in human early embryos. Then, we reveal that paternally expressed ZNF675 is essential for human ZGA. Interestingly, ZNF675 is a primate specific TF with KRAB domain. It suggests that the emergence of ZNF675 may contribute to the desynchrony of ZGA from paternal and maternal genomes in human. Previous work has shown that ZNF675 can bind to transposon elements (TEs), such as ERVs, to repress their activities^43,44^ (Supplementary Fig. S10a). In this study, we find that ZNF675 is dramatically activated from the 4-cell to 8-cell stages in human embryos. Therefore, we speculate that ZNF675 may play important role in the repression of TEs during MZT. To validate our hypothesis, we checked the expression of transposons in human ZNF675 KD embryos at the 8-cell stage. Our data show that many ERVs and L1 elements are elevated upon ZNF675 KD (Supplementary Fig. S10b). It may suggest that the paternally expressed ZNF675 can repress the activities of these transposons during MZT. Consistently, many ERVs and L1 elements, which are not transcribed in human AG embryos at the 8-cell stage, are actively transcribed in PG embryos (Supplementary Fig. S10b). Previous work has revealed that, in Piwil1-deficient embryos, abnormal activation or persistent existence of endogenous retroviruses (ERVs) was associated with impaired ZGA and the decay of maternally deposited transcripts^14^. Thus, the paternally expressed ZNF675 represses transposons at the ZGA stage, which is essential for ZGA and maternal RNA degradation. However, we could not exclude the possibility that ZNF675 can directly regulate the transcription of ZGA genes. LSM1, a RNA-binding protein, is a key component in the LSM1-7 complex. The LSM1-7 complex plays important roles in deadenylation-dependent mRNA decay^37^. Our data show that LSM1-dependent mRNA decay machinery is essential for MRD in human early embryos during MZT. It is well-known that MRD is required for ZGA. Consistently, our data show that ZGA is affected in LSM1 KD 8-cell embryos. Taken together, LSM1 harbors paternally specific promoter DHS and is paternally expressed in human 8-cell embryos. Paternally expressed LSM1 participates in MRD through deadenylation-dependent mRNA decay during MZT, which is required for the normal ZGA.

It is well-known that the failure of ZGA and maternal mRNA decay are correlated with early developmental arrest in the in vitro fertilized human embryos^1,45^, the ZNF675 and LSM1 KD embryos would show developmental arrest. We have checked the developmental abilities of ZNF675 KD and LSM1 KD embryos. For three ZNF675 KD 8-cell embryos, all of them can develop into morulae. However, none of them can develop into blastocysts with blastocyst cavities (Supplementary Fig. S11a, b). For four LSM1 KD 8-cell embryos, all of them can develop into blastocysts (Supplementary Fig. S11a, b). The further developmental potential of LSM1 KD embryos were not assessed due to technical and ethic limitations. These results indicate ZGA defect of ZNF675 and LSM1 KD embryos at the 8-cell stage may be caused by a developmental delay or ZGA delay in the KD embryos.

Our data indicate that the delay of ZGA in PG embryos could be attributed to the epigenomic state of maternal genome, including chromatin accessibility and DNA methylation. During parental pronuclei fusion, the paternal pronucleus is visually larger than the maternal pronucleus. This suggests that the paternal genome is under a less condensed state or a more permissive state than the maternal genome. Consistently, we investigated the DNA methylation and chromatin accessibility patterns in human PG and AG 8-cell embryos. The data show that the global DNA methylation level of AG embryos is lower than that of PG embryo. For chromatin accessibility, although the numbers of DHSs in AG and PG embryos are comparable at the 8-cell stage, the binding motif of TATA-binding protein (TBP), which is a general transcription factor, is enriched in the AG-specific DHSs (Fig. 4c). This result suggests that the paternal genome is more permissive for active transcription than the maternal genome. The relaxing chromatin state in paternal genome is probably a critical factor involved in the initiation of ZGA.

The ZGA delay is not observed in human PG embryos reported in Leng et al. study^32^. The striking differences in manipulation procedures to generate PG and AG embryos may explain the differences in the findings between this and the previous studies. In particular, the PG embryos are generated by activating mature oocyte with Ca^2+^ in Leng et al.’s study, while the PG embryos used in our study are generated by removing male pronucleus before pronuclear fusion in zygotes. In our procedure to generate PG embryos, the factors carried by sperm, such as proteins and small non-coding RNAs, may affect the reprogramming of maternal genome during early embryo development. It can greatly mimic the cellular environments for the reprogramming of maternal genome in diploid embryos. However, in Leng et al.’s study, no paternal factor is introduced into the oocytes and involved in the reprogramming of maternal genome. It may result in that the chromatin states of maternal genome in PG embryos between Leng et al. and our studies are quite distinct. Consequently, during ZGA stages from 4-cell to 8-cell stages, the transcriptomes of PG embryos between Leng et al. and our studies show contradict features. ZGA and MRD are delayed in the PG 8-cell embryos generated in our study, while MBGs were apparent in the PG 4-cell embryos generated in Leng et al.’s study.

Taken together, our data show that paternal genome switches on MRD and ZGA in human embryos at the 8-cell stage. Our data provide a valuable resource in investigating the different roles between paternal and maternal genomes in human early embryos.

## Materials and methods

### Ethics statement

The regulatory framework about the use of human gametes and embryos for this research is based on the policies of the Human Biomedical Research Ethics Guidelines (set by National Health Commission of the People’s Republic of China on Dec. 1st, 2016), the 2016 Guidelines for Stem Cell Research and Clinical Translation issued by the International Society for Stem Cell Research (ISSCR) and the Human Embryonic Stem Cell Research Ethics Guidelines (set by China National Center for Biotechnology Development on Dec. 24th, 2003). These policies and guidelines permit human gametes, and/or human embryos created or genetically manipulated in vitro no more than 14 days, can be used specifically for scientific researches.

The aims and protocols of this study were approved by the Institutional Review Board of Reproductive Medicine of Shandong University (201810). The human gametes used in this study were donated by the patients under assisted reproductive therapy after they signed the informed consents. They were informed that the donation would not affect the process of their therapy.

### Collection of human haploid embryos

The parthenogenetic (PG) and androgenetic (AG) embryos were generated as described^30,31^. No statistical methods were used to predetermine sample size. The immature oocytes at metaphase-I (MI) or germinal vesicle (GV) phase were donated by the patients who were receiving the in vitro fertilization (IVF) treatment. The immature oocytes were cultured in vitro to metaphase-II (MII) stage in IVM culture medium for 24–28 h. The remaining sperm from the donors after their IVF treatment was collected for intracytoplasmic sperm injection (ICSI). The IVM oocytes were fertilized by using ICSI, and then cultured in a time-lapse incubator (EmbryoScope, Vitrolife) for 4–8 h. The maternal and paternal pronucleus can be distinguished by the time-lapse imaging as the maternal pronucleus comes from the second polar body. Next, the zygotes were used for the generation of haploid embryos.

To generate the androgenetic haploid embryos, the second polar body and the maternal pre-pronucleus were removed by using the Blastomere Bilpsy pipette (Sunlight Medical). The zygotes with only paternal genome were termed as human androgenic haploid embryos. The androgenic embryos were then cultured in G-1^TM^ PLUS medium (Vitrolife, 10128) (6% CO_2_, 5% O_2_, 37 °C) to 8-cell stage and then transferred into G-2^TM^ PLUS medium (10132, Vitrolife) for further culturing to blastocyst stage.

To generate the parthenogenetic haploid embryos, the paternal pronucleus was removed by using ICSI injection pipette (Sunlight Medical). The zygotes with only maternal genome were termed as human pathogenic haploid embryos. The parthenogenetic embryos were then cultured in G-1^TM^ PLUS medium (6% CO_2_, 5% O_2_, 37 °C) to eight-cell stage and then transferred into G-2^TM^ PLUS medium for further culturing to blastocyst stage.

After removing the zona pellucida, the AG and PG embryos with high qualities were collected for further experiments.

### Collection of human diploid embryos

The donated IVM oocytes were in vitro fertilized in G-IVF^TM^ PLUS medium (Vitrolife, 10136) in a humidified atmosphere at 37 °C with 6% CO_2_ in air. Cultured 4-cell, 8-cell embryos, morula and morphological AA grade blastocysts were collected around 48 h, 3 days, 4 days and 5 days after fertilization, respectively. Only high-quality embryos were selected and collected for further experiments.

### Quantitative PCR (qPCR)

A single human embryo or one hundred HEK 293T cells were lysed in 2 μL lysis buffer (0.2% Triton X-100, 2 U/μL RNase inhibitor), followed by reverse transcription with SuperScript II reverse transcriptase (Invitrogen, 18064-014). The obtained cDNA was applied to quantitative PCR by using ChamQ Universal SYBR qPCR Master Mix (Vazyme, Q711-02) on the LightCycler 480 II Instrument (Roche). The primers used were listed as follow.

Human ZNF675: CTGGACACTGCACAGCGGAATT and TGTCTCTTCACAGTCAAAGGCTC.

Human beta-actin: CTGGACACTGCACAGCGGAATT and TGTCTCTTCACAGTCAAAGGCTC. The 2^−ΔΔCT^ method was utilized to calculate the relative expression levels of ZNF675 to beta-actin.

### Western blot

A well of HEK 293T cells were lysed directly in 20 μL 1× SDS-PAGE loading buffer. The samples were run on FuturePAGE 4%–12% Gels (ACE, F11412Gel) and transferred to 0.2 μm nitrocellulose membrane (Bio-Rad). The primary antibodies used were rabbit anti-human ZNF675 antibody (Novus Biologicals, #NBP1-79700) and rabbit anti-GAPDH antibody (Proteintech, 10494-1-AP). Horseradish enzyme labeled goat anti-rabbit IgG (H+L) antibody (ZSGB-Bio, ZB-2301) was used as the secondary antibody. The protein signal was detected by using Immobilon Western Chemiluminescent HRP Substrate (Millipore, WBKLS0500) with Tanon 5200 Chemiluminescent Imaging System.

### RNA-seq library preparation

The human embryos were lysed directly and prepared for cDNA synthesis by using SMART-Seq v4 Ultra Low Input RNA Kit for Sequencing (Takara, 634888). Briefly, the sample volume of human embryos was adjusted to 9.5 μL by adding nuclease-free water. After adding 1 μL 10× Reaction Buffer (0.95 μL 10× Lysis Buffer, 0.05 μL RNase Inhibitor), samples were incubated at room temperature for 5 min, then placed on ice. 2 μL 3′ SMART-Seq CDS Primer II A (12 μM) was added. Following incubation at 72 °C for 3 min, samples were placed on ice for 2 min. cDNA synthesis reaction was initiated by adding 4 μL 5× Ultra Low First-Strand Buffer, 1 μL SMART-Seq v4 Oligonucleotide (48 μM), 0.5 μL RNase Inhibitor (40 U/μL) and 2 μL SMARTScribed Reverse Transcriptase. The reaction was carried out in a thermal cycler with the program: 42 °C for 90 min, 70 °C for 10 min, 4 °C forever. The first-strand cDNA product was amplified by adding 25 μL 2× SeqAmp PCR Buffer, 1 μL PCR Primer II A (12 μM), 1 μL SeqAmp DNA Polymerase and 3 μL nuclease-free water. 16 rounds PCR amplification was employed with the following program: 95 °C for 1 min; 98 °C for 10 s, 65 °C for 30 s and 68 °C for 3 min, repeat these 3 steps for 15 times; 72 °C for 10 min; 4 °C forever. The amplified cDNA was purified using 1 volume of SPRIselect beads (Beckman Coulter, B23318), then fragmented to 200–400 bp by Covaris sonicator (Covaris). Sequencing libraries were prepared with NEBNext Ultra II DNA Library Prep Kit for Illumina (NEB, E7645S) according to manufactory’s instruction. In order to obtain adequate amount of DNA for sequencing, the cycle of PCR amplification was determined according to the DNA amount in 1 μL amplified DNA, which was evaluated by using FlashGel System (Lonza, 57063). The libraries were sequenced as 150 bp paired-end on the HiSeq X-Ten platform (Illumina). For each RNA-seq assay, one or several haploid or diploid embryos were used. At least two biological replicates were carried out for PG and AG embryos at 4-cell, 8-cell, morula and blastocyst stages.

### PBAT library preparation

PBAT experiments were performed as described with some modifications^46,47^. Briefly, human haploid embryos were firstly lysed in the lysis buffer (20 mM Tris-HCl pH 8.0, 2 mM EDTA, 20 mM KCl, 2 mg/mL proteinase K) at 56 °C for 1.5 h, followed by heat-inactivation at 75 ^o^C for 30 min. Bisulfite treatment was performed by using EZ DNA Methylation-Gold Kit (Zymo Research, D5006) according to manufactory’s instruction. Next, the first-strand DNA for bisulfite treated DNA was synthesized by using 75 U of Klenow Fragment (3′-5′ exo-) (NEB, M0212M) with the biotinylated random primer BioPEA_N4_37 (5′-biotin-ACA CTC TTT CCC TAC ACG ACG CTC TTC CGA TCT NNN N-3′). This random priming and extension were repeated for five times in total. After that, the excessive primers were removed by the incubation with 40 U of Exonuclease I (NEB, M0293S) at 37 °C for 1 h, followed by the DNA purification with 1 volume of SPRIselect beads. The biotinylated DNA was captured by using Streptavidin beads. The second-strand DNA was synthesized by using 75 U of Klenow Fragment (3′-5′ exo-) with another random primer 2.0-N (5′-GTG ACT GGA GTT CAG ACG TGT GCT CTT CCG ATC TNN NN-3′). Then, DNA fragments bound to Streptavidin beads were amplified by 10–15 cycles of PCR with the primers for the Illumina TruSeq DNA libraries. Lastly, the DNA fragments of sizes from 300 to 700 bp were purified with SPRIselect beads. The libraries were sequenced as 150 bp paired-ends on the HiSeq X-Ten instrument (Illumina). For a PBAT library, four haploid 8-cell embryos were used. At least two biological replicates were performed for PG and AG embryos.

### DNase-seq library preparation

DNase-seq experiments were performed as described with some modifications^7^. Briefly, embryos were lysed in 40 μL of cold lysis buffer (10 mM Tris-HCl pH 7.5, 10 mM NaCl, 3 mM MgCl_2_, 0.5% Triton X-100) on ice for 30 min. 10 μL of diluted DNaseI (Roche, 04716728001) was added to the final concentration of 150 U/mL and incubated at 37 °C for 5 min. Reaction was stopped by adding 50 μL stop buffer (10 mM Tris-HCl pH 7.5, 10 mM NaCl, 0.2% SDS, 20 mM EDTA) containing 40 μg Proteinase K (Qiagen, 19133) followed by incubation at 55 °C for 1 h. After adding 300 ng carrier RNA (Tiagen, RT416-02), the DNA was purified by Zymo Oligo Clean Concentrator (Zymo Research, D4060) and then eluted in 50 μL TE (2.5 mM Tris-HCl pH 7.5, 0.05 mM EDTA). The DNA libraries were constructed by using NEBNext Ultra II DNA Library Prep Kit for Illumina (NEB, E7645S) according to manufactory’s instruction. After 8 cycles of PCR amplification, the DNA fragments of sizes from 150 to 400 bp were selected with 0.7 volume plus 0.7 volume of SPRIselect beads (Beckman Coulter, B23318). The DNA products were amplified by another 7 cycles of PCR, followed by purification with 1.3 volume of SPRIselect beads. The libraries were sequenced as 150 bp paired-ends on the HiSeq X-Ten platform (Illumina). For a DNase-seq library, five haploid 8-cell embryos were used. Two biological replicates were carried out for PG and AG embryos at each stage.

### α-amanitin treatment

The PG or AG zygotes were cultured in G-1^TM^ PLUS medium with 25 ng/μL α-amanitin (Sigma-Aldrich). The treated PG or AG embryos with high qualities were collected at the 8-cell stage for RNA-seq experiments.

### Gene knockdown by siRNA injection

To investigate the functions of ZNF675, LSM1, and EXOSC5 in maternal RNA clearance and ZGA, the siRNAs targeting human ZNF675, LSM1, and EXOSC5 were synthesized for microinjection. The sequences of siRNAs are listed below:

ZNF675 siRNA #1 (GAGCCUUUGACUGUGAAGATT)

ZNF675 siRNA #2 (CCUAACUCGACAUGAAAGATT)

ZNF675 siRNA #3 (GCUCGAGAGAAACCAUACATT)

LSM1 siRNA #1 (GCCAGCCUCAUCGAGGACATT)

LSM1 siRNA #2 (GCAAGUAUCCAUUGAAGAATT)

LSM1 siRNA #3 (CCUGAAGGACCGAGGUCUUTT)

EXOSC5 siRNA #1 (CCUGGCCUGUUGUCUGAAUTT)

EXOSC5 siRNA #2 (GGAUCCUACAUCCAAGCAATT)

EXOSC5 siRNA#3 (CCACACUCGAAGUGAUCCUTT)

Individual ZNF675 siRNA was used to knockdown ZNF675. In addition, the mixture of ZNF675 siRNAs #1-#3, LSM1 siRNAs #1-#3 and EXOSC5 siRNA #1-#3 were used to knockdown ZNF675, LSM1 and EXOSC5 respectively. The final concertation of each siRNA was 20 μM.

The donated IVM oocytes were in vitro fertilized and cultured in G-IVF^TM^ PLUS medium. siRNA solution was injected into the zygote before pronuclear fading by using Eppendorf PiezoXpert and Eppendorf CellTram vario microinjector. The injected embryos were cultured in G-1^TM^ PLUS medium in a humidified atmosphere at 37 °C with 6% CO_2_ in air. The injected embryos with normal morphology were harvested at 8-cell stage 3 days after fertilization. The embryos injected with water were used as control. One 8-cell embryo was used for a RNA-seq library. Three biological replicates were performed for each group.

### RNA-seq data analysis

The sequence reads were trimmed by using Trimmomatic v0.39 to remove adapter sequence and the reads with low qualities^48^. Paired reads were mapped to the human genome (version hg19) by hisat2 v2.1.0^49^ and to the transcriptome by Salmon v 0.8.2^50^. To quantify the gene expression levels, the FPKM values of genes were calculated by using Cufflinks-2.2.1^51^. The DESeq2 v1.18.0 software^52^ was used to identify differentially expressed genes (DEGs) between AG and PG haploid embryos from 4-cell to blastocyst stages, based on the raw counts of reads in genes, which was produced by Salmon. The DEGs should satisfy two criteria: the adjusted *P* value < 0.05, and the fold change of gene expression level > 2. Furthermore, all the replicates for the embryos in a group were combined and the FPKM values of gene expression were re-calculated by using Cufflinks 2.2.1. All DEGs between AG and PG haploid embryos were further filtered as DEGs should show FPKM values ≥ 1 in either PG or AG embryos. The X chromosome DEGs related with AG morulae were not counted due to there was no X chromosome in AG morulae used in RNA-seq. Metascape^53^ was used to perform Gene Ontology (GO) and KEGG analysis for the specific DEGs. The spearman correlation between replicates (R1, R2, or R3) were calculated by using the “cor” function in R. The tracks for the normalized RNA-seq signal, which was indicated as the read number per million reads on the covered genomic position, were generated by using the “genomecov” in bedtools suit and “bedGraphToBigwig” tool.

### PBAT DNA methylome data analysis

The sequence reads were trimmed by using Trimmomatic v0.39 to remove adapter sequence and the reads with low qualities were discarded. Paired reads were mapped to the human genome (version hg19) by using Bismark_v0.20.0^54^. The paired reads failed to align to human genome were re-aligned to the genome in the single-end mode. Duplicated reads were removed by deduplicate_bismark tool in Bismark. The overlapped region in the genome between a pair of reads was clipped from one read by using clipOverlap function in bamUtil. Both paired-end alignment and single-end alignment were combined to calculated CpG methylation level (ML) for each CpG site. Only the CpG sites with read depth ≥ 3 were kept for further analysis.

### Quantification of methylation levels of CpGs and genomic elements

For a CpG site i, we defined m_i_ as the number of reads showing methylation at cytosines (methylated Cs) in both strands. We defined u_i_ as the number of reads showing unmethylation at cytosines (unmethylated Cs) in both strands. The methylation level of the CpG site i is estimated as m_i_/(m_i_ + u_i_). The methylation level of a genomic element was calculated as the ratio of the number of methylated Cs to all of the methylated and unmethylated Cs in the genomic elements. Promoters are referred to the regions from 1 kb upstream to 1 kb downstream of TSSs (transcriptional start sites) (TSS ± 1 kb). Only the promoters with at least 5 different CpG sites were covered by sequencing reads were considered for further analysis.

### Identification of the differentially methylated sites/regions (DMSs/DMRs) between AG and PG embryos

The CpG sites with read depth ≥ 5 were considered for statistical test. The differences of the methylation levels of CpG sites between two samples was evaluated by a two-tailed Fisher’s Exact Test. The *P* values were adjusted by the method of Benjamini and Hochberg^55^. The CpG sites with the adjusted *P* values < 0.1 were defined as DMSs. If not specified, the methylation level differences of all DMSs between two samples should be greater than 0.2. Then, maternal DMSs (higher CpG methylation in PG embryos) were merged into maternal pre-DMRs if the distances between adjacent maternal DMSs were less than 500 bp. Paternal DMSs (higher CpG methylation in AG embryos) were merged into paternal pre-DMRs if the distances between adjacent paternal DMSs were less than 500 bp. Next, the maternal and paternal pre-DMRs were combined into pre-DMRs. The final DMRs were obtained if the pre-DMRs contained at least three DMSs, adjusted *P* values were less than 0.1, the methylation level differences of pre-DMRs must be greater than 0.2, and the regions sizes were larger than 50 bp.

### DNase-seq data analysis

The sequencing reads were cropped to 100 bp from 3′ end and trimmed by using Trimmomatic v0.39 to remove adapter sequence and reads with low qualities^48^. All Reads 1, and Reads 2 from unpaired reads were aligned to human genome hg19 by Bowtie v1.2.0^56^ with parameter “-m 1”. The PCR duplicated reads were removed by Picard v2.18.25. DHSs were called by hotspot algorithm with FDR < 0.01^57^. The Fragment Per Kilobase per Million mapped reads (FPKM) value for each genome-wide non-overlapped 5 kb window was calculated as tag density for DNase-seq data. The Pearson correlation coefficient (*r*) of tag densities in 5 kb bins between two biological replicates was used to evaluate the reproducibility. Two biological replicates were merged to call DHS peaks. To obtain DHS master list, we concatenated DHSs in PG and AG embryos at 8-cell and blastocyst stages, the overlapped DHSs were merged into a large DHS. The DHSs in master list overlapping with original DHSs in PG or AG embryos were defined as DHSs in such samples for further analysis. The FPKM values of DHSs in each sample were calculated. The tracks of DNase-seq signal visualized in Integrative Genomics Viewer (IGV) were generated by bamCoverage in Deeptools2 suite with parameters “--noralizeUsingRPKM --extendReads 150”^58–60^. Due to different signal-to-noise ratio between PG and AG samples at the same developmental stage, the sequencing depth normalized DHS signal was not suitable to be used directly to call allelic DHSs between PG and AG samples. As described in previous work^61^, we assumed that the top 10% DHSs (5000–10,000 DHSs) should show similar signal levels between PG and AG samples. Thus, we calculated the scale factor by the ratio of median signal level in the top 10% DHS peaks between PG and AG samples. Finally, the FPKM value for each DHS was multiplied by the scale factor for comparison. After scale factor normalization, the DHSs that were only detected in AG embryos and showed signal fold change (AG/PG) more than 2 were defined as AG-specific DHSs, vice versa were PG specific DHSs.

### Allelic RNA expression in mouse embryos

To analyze the gene expression pattern parental genomes in mouse early embryos, we downloaded the RNA-seq data from GSE71434. The SNP information among common mouse strains for mm10 genome version is downloaded from Mouse Genomes Project. SNPsplit was used to distinguish maternal and paternal reads in RNA-seq data for mouse late 2-cell and ICM. For each gene covered by reads with parental SNPs, we counted the numbers of reads from paternal and maternal genomes, respectively. The reads numbers were then checked by Binomial test. For a gene with bi-allelic expression, the probabilities of a sequencing read from paternal genome or maternal genome are both 0.5. A gene with binomial test *P* value < 0.001 (BH adjusted *P* value < 0.05) and the fold change of reads numbers between parental genomes > 2 was considered as an allelically expressed gene. In addition to SNP-based allelic expression, we also generated mouse AG and PG late 2-cell embryos to examine the expression difference of parental genomes. The methods to generated mouse AG and PG embryos were similar to human haploid embryos.

### Human ZGA genes and maternal degradation

The list of human ZGA genes in diploid embryos were obtained from previous study^15^, with additional selection that the ZGA genes show expression in 8-cell in this study. The maternally degraded genes at 8-cell stage from haploid 4-cell to 8-cell were those 4-cell AG or PG biased DEGs comparing to 8-cell AG or PG, with the FPKM ≥ 2 in 4-cell AG or PG. The newly transcribed genes from haploid 4-cell to 8-cell were those 8-cell AG or PG biased DEGs comparing to 4-cell AG or PG, with the FPKM ≥ 2 in 8-cell AG or PG.

### Quantification of parental gene expression using traceable loci

The paternal exome-seq and embryos RNA-seq data were from^35^, and trimmed using Trimmomatic and aligned to hg19 using hisat2 v2.1.0 with default parameters. Base information for each gene were obtained using “samtools mpileup”, only sites (loci) which were homozygous in exome-seq and covered by more than 10 reads in both exome-seq and RNA-seq, were taken for further parental-ratio calling. In RNA-seq of embryos, if these sites contained bases that were different from paternal bases, we inferred the alternative bases were maternally derived, and reads containing these bases are maternally expressed. Only sites were taken into consideration and regarded as SNPs if at least 5% reads could be detected from the alternative bases and at least three reads covered the alternative bases.

### Quantification of the expression of retrotransposons in human embryos

Homer^62^ was used to calculated the gene expression levels (FPKM) of retrotransposons which were downloaded from RepeatMasker. For each subfamily of retrotransposons with PG specifically high expression, the expression levels (FPKM) in PG embryos are more than 20, and at least twice as high as the gene expression levels (FPKM) in AG embryos. For each subfamily of retrotransposons with AG specifically high expression, the expression levels (FPKM) in AG embryos are more than 20, and at least twice as high as the gene expression levels (FPKM) in PG embryos.

### Cluster analysis

PCA analysis of all samples based on their RNA expression patterns were performed by using “princomp” function in R (v3.4.4). K-mean analysis were performed by using the “Cluster 3.0” software. The heatmaps were drawn by ggplot2. Hierarchical clustering was done by using the “cluster” function with the “ward.D” method for human samples, with the “complete” method for mouse samples, based on the log transformed RNA expression levels.

### Annotation files for genomic elements

The hg19 refGene files downloaded from UCSC Table Browser were used for genome annotations. The annotation files for genomics elements, including promoters (TSS ± 1 kb), exons and introns, were downloaded from UCSC Table Browser.

### Statistical analysis

Statistical analyses and plots were implemented with R(3.4.4) (http://www.r-project.org). Pearson or Spearman Correlation Coefficients were calculated by using the ‘cor’ function. Wilcoxon rank sum test was performed by using ‘wilcox.test’ function (two.sided). Hypergeometric test was performed by using ‘phyper’ function.

## Supplementary information

Supplementary Information, Figs S1-S11

Supplementary Table S1

Supplementary Table S2

Supplementary Table S3

Supplementary Table S4

Supplementary Table S5

## Data Availability

Human sequencing data generated in this study have been deposited in the Genome Sequence Archive (GSA) with the accession number HRA000888. Mouse sequencing data generated in this study have been deposited in the Genome Sequence Archive (GSA) with the accession number CRA005750. In addition, external sequencing data used in this study are listed below: CUT&RUN of H3K27me3 in 8-cell (GSE124718), RNA-seq in OCT4 KD (siRNA) and WT human 8-cell (GSA: CRA000297), RNA-seq and exome-seq in human blood and embryos (GSE44183, GSE36552), RNA-seq in mouse embryos (GSE71434, CRA000108). ZNF675 ChIP-seq in HEK 293 (GSE58341) and ZNF675 ChIP-exo in HEK 293T (GSE78099). All codes are available upon reasonable request.

## References

[1] Sha QQ (2020). Dynamics and clinical relevance of maternal mRNA clearance during the oocyte-to-embryo transition in humans. Nat. Commun..

[2] Sha QQ (2020). Characterization of zygotic genome activation-dependent maternal mRNA clearance in mouse. Nucleic Acids Res..

[3] Tadros W, Lipshitz HD (2009). The maternal-to-zygotic transition: A play in two acts. Development.

[4] Vassena R (2011). Waves of early transcriptional activation and pluripotency program initiation during human preimplantation development. Development.

[5] Schulz KN, Harrison MM (2019). Mechanisms regulating zygotic genome activation. Nat. Rev. Genet..

[6] Lee MT, Bonneau AR, Giraldez AJ (2014). Zygotic genome activation during the maternal-to-zygotic transition. Annu. Rev. Cell Dev. Biol..

[7] Gao L (2018). Chromatin accessibility landscape in human early embryos and its association with evolution. Cell.

[8] Wu J (2018). Chromatin analysis in human early development reveals epigenetic transition during ZGA. Nature.

[9] Lu F (2016). Establishing chromatin regulatory landscape during mouse preimplantation development. Cell.

[10] Vuoristo S (2022). DUX4 is a multifunctional factor priming human embryonic genome activation. iScience.

[11] Macfarlan TS (2012). Embryonic stem cell potency fluctuates with endogenous retrovirus activity. Nature.

[12] Percharde M (2018). A LINE1-nucleolin partnership regulates early development and ESC identity. Cell.

[13] Svoboda P (2004). RNAi and expression of retrotransposons MuERV-L and IAP in preimplantation mouse embryos. Dev. Biol..

[14] Zhang HD (2021). The piRNA pathway is essential for generating functional oocytes in golden hamsters. Nat. Cell Biol..

[15] Chen X (2019). Key role for CTCF in establishing chromatin structure in human embryos. Nature.

[16] Li C (2018). DNA methylation reprogramming of functional elements during mammalian embryonic development. Cell Discov..

[17] Smith ZD (2014). DNA methylation dynamics of the human preimplantation embryo. Nature.

[18] Xia W (2019). Resetting histone modifications during human parental-to-zygotic transition. Science.

[19] Wu J (2016). The landscape of accessible chromatin in mammalian preimplantation embryos. Nature.

[20] Ke Y (2017). 3D chromatin structures of mature gametes and structural reprogramming during mammalian embryogenesis. Cell.

[21] Plasschaert RN, Bartolomei MS (2014). Genomic imprinting in development, growth, behavior and stem cells. Development.

[22] Wang L (2014). Programming and inheritance of parental DNA methylomes in mammals. Cell.

[23] Chen ZY, Yin QZ, Inoue A, Zhang CX, Zhang Y (2019). Allelic H3K27me3 to allelic DNA methylation switch maintains noncanonical imprinting in extraembryonic cells. Sci. Adv..

[24] Du Z (2017). Allelic reprogramming of 3D chromatin architecture during early mammalian development. Nature.

[25] Miller RD (2005). High-density single-nucleotide polymorphism maps of the human genome. Genomics.

[26] Leeb M, Wutz A (2011). Derivation of haploid embryonic stem cells from mouse embryos. Nature.

[27] Tarkowski AK, Rossant J (1976). Haploid mouse blastocysts developed from bisected zygotes. Nature.

[28] Yang H (2012). Generation of genetically modified mice by oocyte injection of androgenetic haploid embryonic stem cells. Cell.

[29] Inoue A, Jiang L, Lu F, Suzuki T, Zhang Y (2017). Maternal H3K27me3 controls DNA methylation-independent imprinting. Nature.

[30] Zhang XM (2020). In vitro expansion of human sperm through nuclear transfer. Cell Res..

[31] Zhong CQ (2016). Generation of human haploid embryonic stem cells from parthenogenetic embryos obtained by microsurgical removal of male pronucleus. Cell Res..

[32] Leng L (2019). Single-cell transcriptome analysis of uniparental embryos reveals parent-of-origin effects on human preimplantation development. Cell Stem Cell.

[33] Zhang B (2016). Allelic reprogramming of the histone modification H3K4me3 in early mammalian development. Nature.

[34] Yan L (2013). Single-cell RNA-Seq profiling of human preimplantation embryos and embryonic stem cells. Nat. Struct. Mol. Biol..

[35] Xue Z (2013). Genetic programs in human and mouse early embryos revealed by single-cell RNA sequencing. Nature.

[36] Jiang, Z. Y. & Fan, H. Y. Five questions toward mRNA degradation in oocytes and preimplantation embryos: When, who, to whom, how, and why? *Biol. Reprod.* **107**, 62–75 (2022). 10.1093/biolre/ioac014 35098307

[37] Garneau NL, Wilusz J, Wilusz CJ (2007). The highways and byways of mRNA decay. Nat. Rev. Mol. Cell Biol..

[38] Yadav T, Quivy JP, Almouzni G (2018). Chromatin plasticity: A versatile landscape that underlies cell fate and identity. Science.

[39] Shi G, Jin Y (2010). Role of Oct4 in maintaining and regaining stem cell pluripotency. Stem Cell Res. Ther..

[40] Bentsen M (2020). ATAC-seq footprinting unravels kinetics of transcription factor binding during zygotic genome activation. Nat. Commun..

[41] Liu X (2016). Distinct features of H3K4me3 and H3K27me3 chromatin domains in pre-implantation embryos. Nature.

[42] Zheng H (2016). Resetting epigenetic memory by reprogramming of histone modifications in mammals. Mol. Cell.

[43] Garton M (2015). A structural approach reveals how neighbouring C2H2 zinc fingers influence DNA binding specificity. Nucleic Acids Res..

[44] Imbeault M, Helleboid PY, Trono D (2017). KRAB zinc-finger proteins contribute to the evolution of gene regulatory networks. Nature.

[45] Yu X (2022). Recapitulating early human development with 8C-like cells. Cell Rep..

[46] Miura F, Enomoto Y, Dairiki R, Ito T (2012). Amplification-free whole-genome bisulfite sequencing by post-bisulfite adaptor tagging. Nucleic Acids Res..

[47] Smallwood SA (2014). Single-cell genome-wide bisulfite sequencing for assessing epigenetic heterogeneity. Nat. Methods.

[48] Bolger AM, Lohse M, Usadel B (2014). Trimmomatic: A flexible trimmer for Illumina sequence data. Bioinformatics.

[49] Pertea M, Kim D, Pertea GM, Leek JT, Salzberg SL (2016). Transcript-level expression analysis of RNA-seq experiments with HISAT, StringTie and Ballgown. Nat. Protoc..

[50] Patro R, Duggal G, Love MI, Irizarry RA, Kingsford C (2017). Salmon provides fast and bias-aware quantification of transcript expression. Nat. Methods.

[51] Trapnell C (2010). Transcript assembly and quantification by RNA-Seq reveals unannotated transcripts and isoform switching during cell differentiation. Nat. Biotechnol..

[52] Love MI, Huber W, Anders S (2014). Moderated estimation of fold change and dispersion for RNA-seq data with DESeq2. Genome Biol..

[53] Zhou Y (2019). Metascape provides a biologist-oriented resource for the analysis of systems-level datasets. Nat. Commun..

[54] Krueger F, Andrews SR (2011). Bismark: A flexible aligner and methylation caller for Bisulfite-Seq applications. Bioinformatics.

[55] Hochberg Y, Benjamini Y (1990). More powerful procedures for multiple significance testing. Stat. Med..

[56] Langmead B, Trapnell C, Pop M, Salzberg SL (2009). Ultrafast and memory-efficient alignment of short DNA sequences to the human genome. Genome Biol..

[57] John S (2011). Chromatin accessibility pre-determines glucocorticoid receptor binding patterns. Nat. Genet..

[58] Ramirez F (2016). deepTools2: A next generation web server for deep-sequencing data analysis. Nucleic Acids Res..

[59] Thorvaldsdottir H, Robinson JT, Mesirov JP (2013). Integrative Genomics Viewer (IGV): high-performance genomics data visualization and exploration. Brief. Bioinform..

[60] Robinson JT (2011). Integrative genomics viewer. Nat. Biotechnol..

[61] Dahl JA (2016). Broad histone H3K4me3 domains in mouse oocytes modulate maternal-to-zygotic transition. Nature.

[62] Heinz S (2010). Simple combinations of lineage-determining transcription factors prime cis-regulatory elements required for macrophage and B cell identities. Mol. Cell.

