# Supplementary material for: Human zygotic genome activation is initiated from paternal genome
Source: Cell Discov. 2023 Jan 31;9:13. doi: 10.1038/s41421-022-00494-z (PMC9887001; doi:10.1038/s41421-022-00494-z)
Supplement: Supplementary file 1 — Supplementary Information, Figs S1-S11 [file 41421_2022_494_MOESM1_ESM.pdf]

Human zygotic genome activation is initiated from paternal genome

Shenli Yuan[1,2,3#], Jianhong Zhan[2,3#], Jingye Zhang[1,4#], Zhenbo Liu[2], Zhenzhen Hou[1,4], Chuanxin Zhang[1,4], Lizhi Yi[2], Lei Gao[2*], Han Zhao[1,4*], Zi-Jiang Chen[1,4,5*], Jiang Liu[2,3,6*], Keliang Wu[1,4*]
[1]Center for Reproductive Medicine, Shandong University, Jinan, Shandong, 250012, China
[2]CAS Key Laboratory of Genome Sciences and Information, Collaborative Innovation Center of Genetics and Development, Beijing Institute of Genomics, and China National Center for Bioinformation, Chinese Academy of Sciences, Beijing 100101, China.
[3]University of Chinese Academy of Sciences, Beijing 100049, China.
[4]Key laboratory of Reproductive Endocrinology of Ministry of Education, Shandong University, Jinan, Shandong, 250012, China.
[5]Center for Reproductive Medicine, Ren Ji Hospital, School of Medicine, Shanghai Jiao Tong University, Shanghai, 200135, China.
[6]CAS Center for Excellence in Animal Evolution and Genetics, Chinese Academy of Sciences, Kunming 650223, China.
[#]These authors contributed equally: Shenli Yuan, Jianhong Zhan, Jingye Zhang.
[*]Corresponding author e-mail: gaol@big.ac.cn; hanzh80@yahoo.com; chenzijiang@hotmail.com; liuj@big.ac.cn; wukeliang_527@163.com.

This file includes:
Supplementary Figs. S1-S11

Additional Files:
Supplementary Table S1: The sample information of human embryos used in RNA-seq, PBAT and DNase-seq.
Supplementary Table S2: GO and KEGG analysis of DEGs between AG and PG embryos.
Supplementary Table S3: The expression of TFs with AG-specific high expression at 8-cell stage and high expression in diploid 8-cell embryo and PG morula.
Supplementary Table S4: GO and KEGG analysis of genes whose promoters overlap with DMRs between AG and PG 8-cell embryos.
Supplementary Table S5: GO and KEGG analysis of genes whose promoters overlap with AG-specific or PG-specific DHSs.

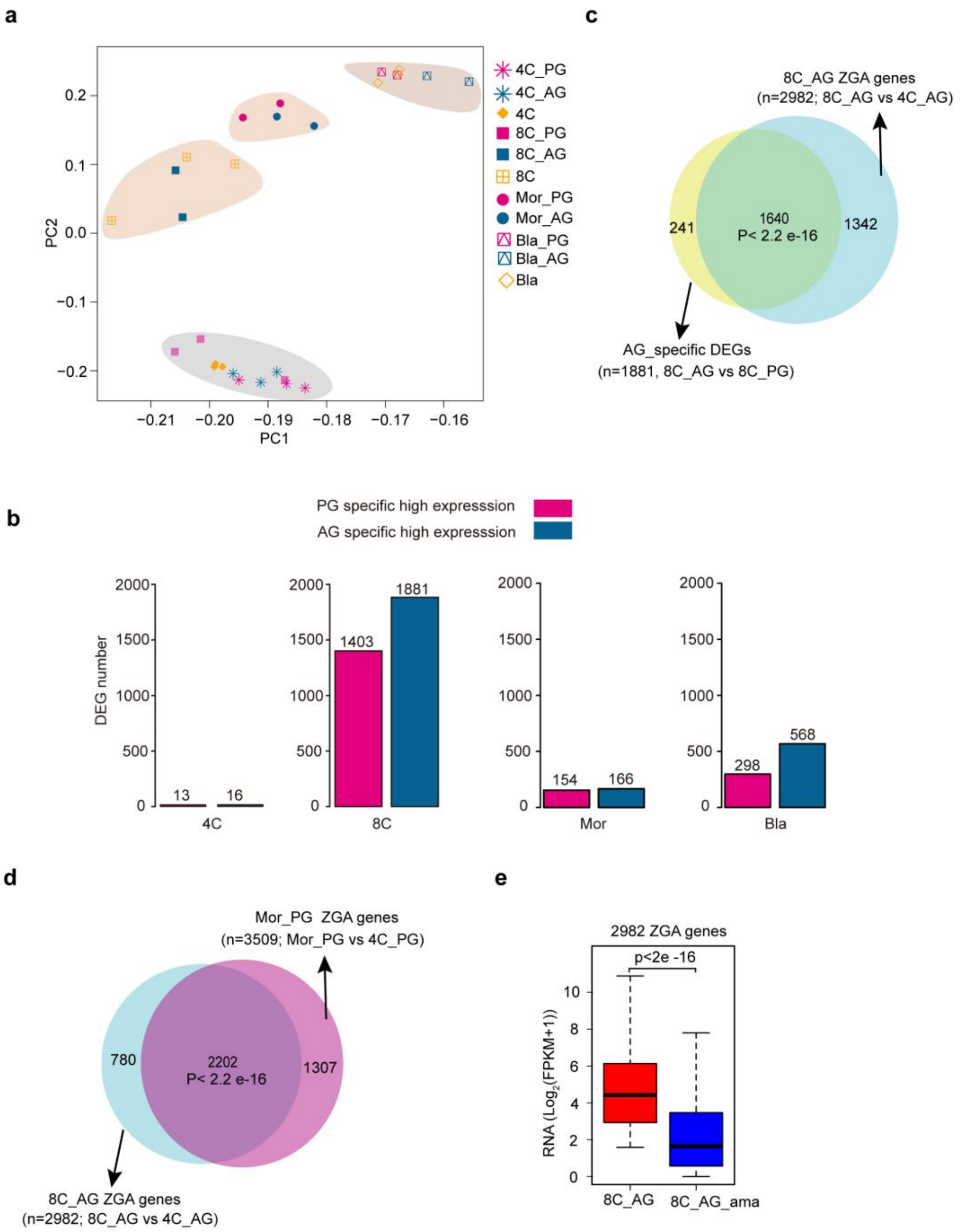

Supplementary Figure S1 ZGA is delayed in human PG 8-cell embryo. a Principal component analysis (PCA) of human haploid and bi-parental embryos according to their transcriptomes. 4C represents 4-cell embryo; 8C represents 8-cell embryo; Mor represents morula; Bla represent blastocyst. **b** Bar plots showing the number of DEGs between PG and AG embryos. The numbers of DEGs are shown above bars. **c** Venn diagram showing the overlapped genes between the AG-specific DEGs at the 8-cell stage and the nascently transcribed genes in AG 8-cell embryo. Hypergeometric test was used. The gene numbers are shown. **d** Venn diagram showing the overlapped genes between the nascently transcribed genes in AG 8-cell embryo (vs AG 4-cell embryo) and those in PG morula (vs PG 4-cell embryo). Hypergeometric test was used. The nascently transcribed genes at the 8-cell stage also referred as ZGA genes. **e** Comparison of RNA expression levels of the nascently transcribed genes in

AG 8-cell embryo (ZGA genes in AG 8-cell embryo) between AG 8-cell embryo and α-amanitin (ama) treated AG 8-cell embryo in human. Wilcoxon rank sum test was used.

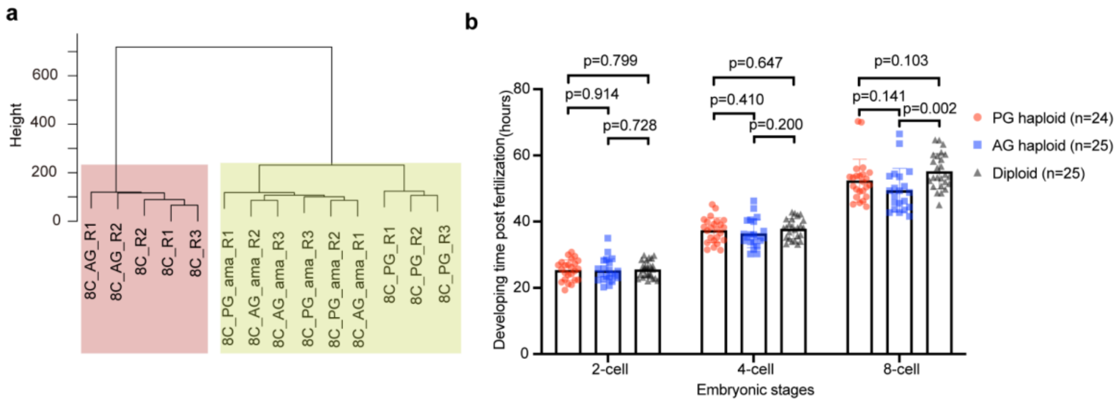

Supplementary Figure S2 The comparison of transcriptomes and developmental time of human haploid and diploid embryos. a Hierarchical clustering of haploid 8-cell embryos, α-amanitin (ama) treated haploid 8-cell embryos and bi-parental 8-cell embryos according to their RNA expression patterns. R1, R2 and R3 represent the 3 biological replicates. **b** Comparison of developmental time of human PG and AG haploid embryos as well as diploid embryos from the 2-cell stage to 8-cell stage after fertilization. t test was used. The developing time for early embryos is calculated from the time point of in vitro fertilization to the time point when the embryo enters the developmental stage. The numbers of embryos in different groups are indicated in in parentheses.

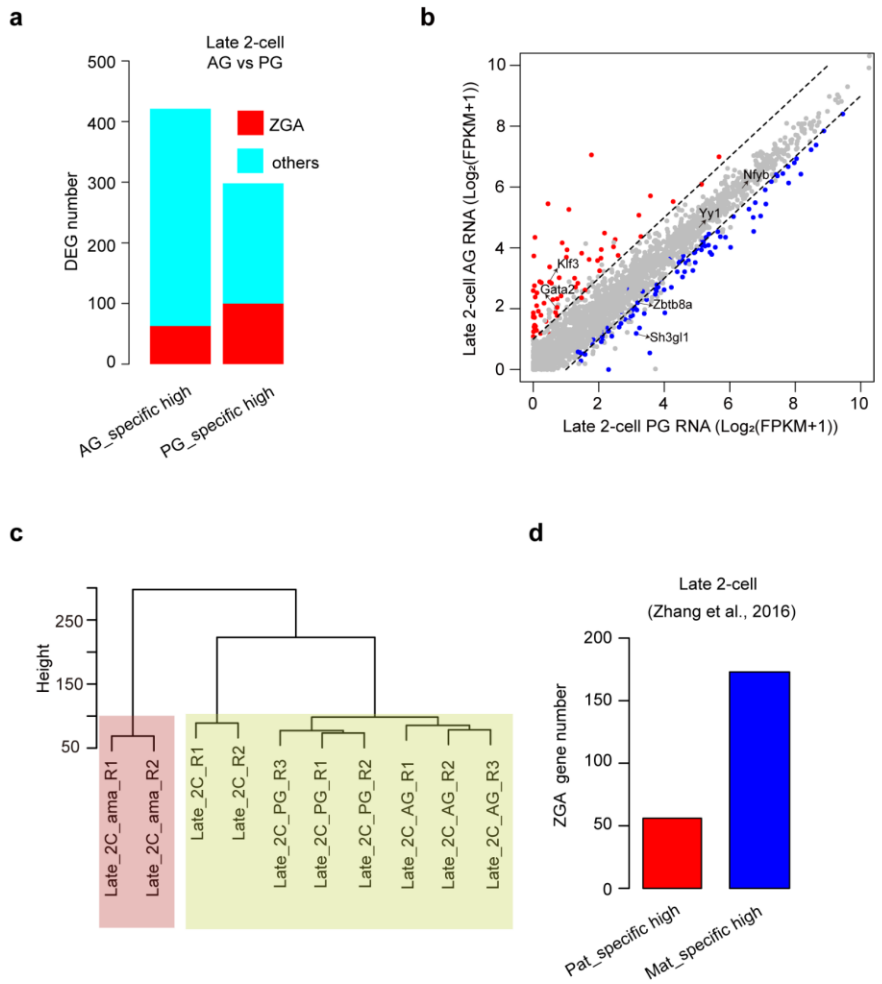

Supplementary Figure S3 Mouse ZGA is comparable between AG and PG embryos at the late 2-cell stage. **a** Bar plot showing the numbers of DEGs in mouse AG and PG late-2cell embryos. The DEGs are classified into two groups, including ZGA genes and others. The mouse ZGA gene list is from (Gao et al., 2018). **b** Scatterplot showing the expression levels of ZGA genes in mouse AG and PG late 2-cell embryos. The genes with PG-specifically high expression are labeled in blue, while the genes with AG-specifically high expression are labeled in red. **c** Hierarchical clustering of mouse late 2-cell AG and PG embryos, bi-parental late 2-cell embryos and α-amanitin (ama) treated late 2-cell embryos according to their RNA expression patterns. R1, R2 and R3 represent the biological replicates. The RNA-seq data of bi-parental late 2-cell embryos and α-amanitin (ama) treated late 2-cell embryos are from (Ke et al., 2017). **d** Boxplot showing the number of ZGA genes with paternally (pat) and maternally (mat) specific expression in mouse late 2-cell embryos. The RNA-seq data are from (Zhang et al., 2016).

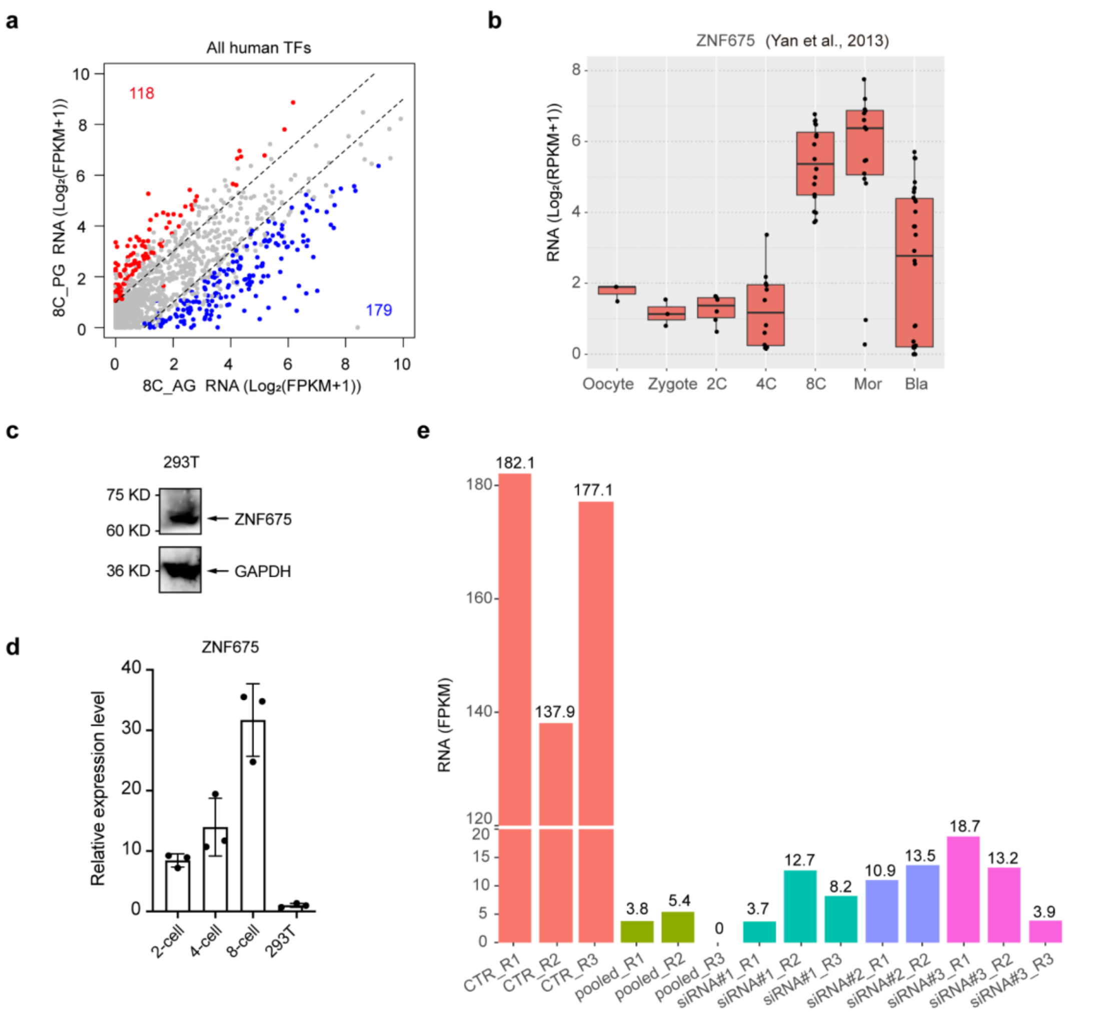

Supplementary Figure S4 The expression pattern of TFs and analysis of DEGs in ZNF675 KD embryos. a Scatter plot showing the expression levels of TFs in human AG and PG 8-cell embryos. The TFs with PG-specifically high expression are labeled in red, while the genes with AG-specifically high expression are labeled in blue. The numbers of TFs belonging to DEGs are indicated. **b** Boxplot showing the gene expression levels of ZNF675 from oocyte to blastocyst. The gene expression of ZNF675 are from published single-cell RNA-seq data (Yan et al., 2013). **c** Western blot of ZNF675 in HEK 293T cells. **d** Quantitative PCR of ZNF675 in human early embryos and HEK 293T cells. The relative expression levels of ZNF675 in human early embryos to that in HEK 293T cells were shown. Error bars represent mean ± standard deviation (SD). **e** The expression levels of ZNF675 among the ZNF675 KD and control (CTR) 8-cell embryos. R1, R2 and R3 represent different biological replicates. siRNA #1, siRNA #2 and siRNA #3 represent three different ZNF675 siRNAs.

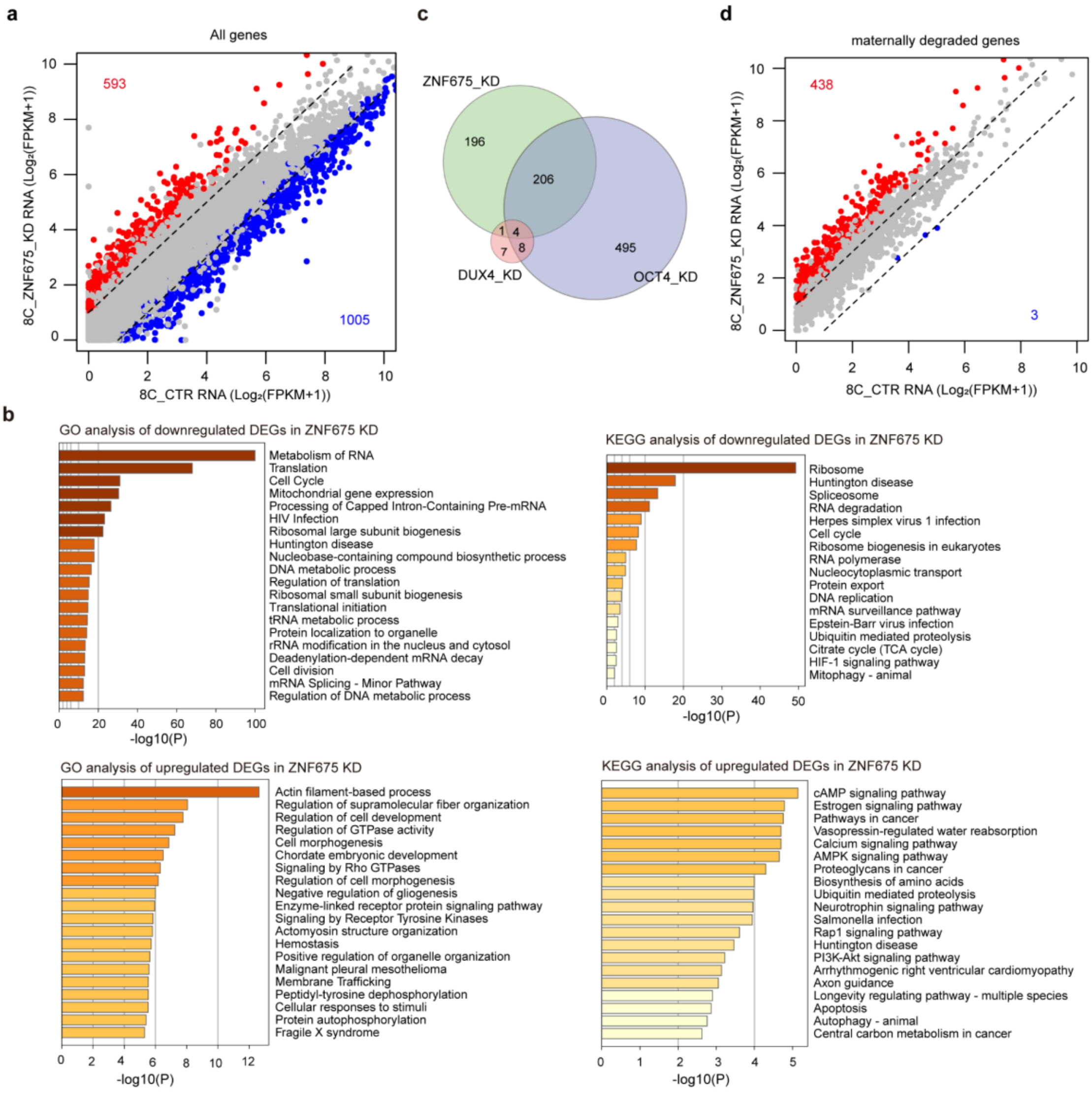

Supplementary Figure S5 The RNA expression and GO analysis of DEGs in ZNF675 KD embryos. a Scatter plot showing the expression levels of genes between ZNF675 KD and control (CTR) 8-cell embryos. The genes upregulated in ZNF675 KD embryos are labeled in red, while the genes downregulated in ZNF675 KD embryos are labeled in blue. The samples from siRNA #1 R1, R2 and siRNA #3 R3 are used as treatment group to investigate the functions of ZNF675 in human early embryo development. The numbers of DEGs are indicated. **b** GO and KEGG analysis of DEGs downregulated in ZNF675 KD embryos (top) and upregulated in ZNF675 KD embryos (bottom). **c** Venn diagram showing the overlap of downregulated ZGA genes among ZNF675 KD, OCT4 KD and DUX4 KD human 8-cell embryos. The downregulated ZGA genes in OCT4 KD embryos are from (Gao et al., 2018). The downregulated ZGA genes in DUX4 KD embryos from (Vuoristo et al., 2022). **d** Scatter plot showing the expression levels of maternally degraded genes between ZNF675 KD and control 8-cell embryos. The genes upregulated in ZNF675 KD embryos are labeled in red, while the genes downregulated in ZNF675 KD embryos are labeled in blue. The numbers of maternally degraded genes belonging to DEGs are indicated.

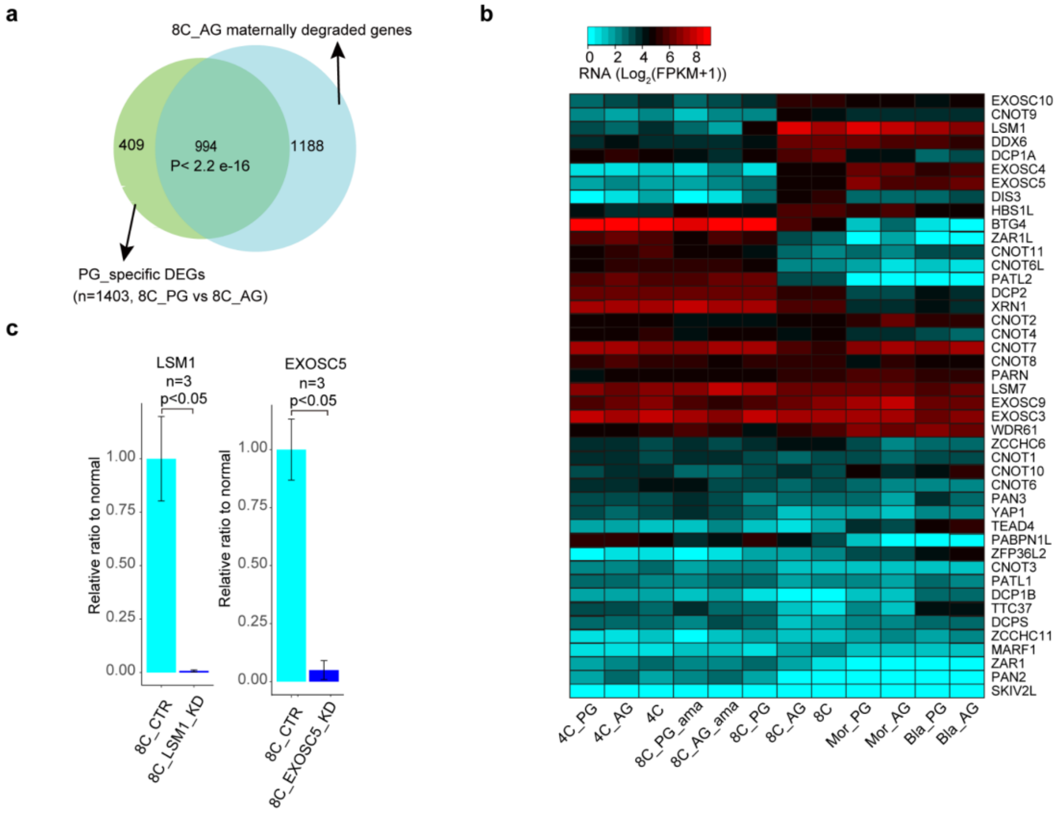

Supplementary Figure S6 The maternal mRNA degradation is delayed in human PG embryo. a Venn diagram showing the overlapped genes between the PG 8-cell specifically expressed genes (vs AG 8-cell embryo) and the maternally degraded genes in AG 8-cell embryo (vs AG 4-cell embryo). Hypergeometric test was used. **b** The expression patterns of mRNA degradation-associated genes in human embryos. The mRNA degradation-associated genes are from (Sha et al., 2020; Jiang et al., 2022; Garneau et al., 2007). **c** Relative expression levels of LSM1 and EXOSC5 in human LSM1 knockdown (KD) and EXOSC5 KD embryos at the 8-cell stage, respectively. The relative ratio represents the ratio of gene expression level in KD group to that in control group (bi-parental embryos). Error bars represent standard errors. t test was used. The numbers (n) of KD samples are shown.

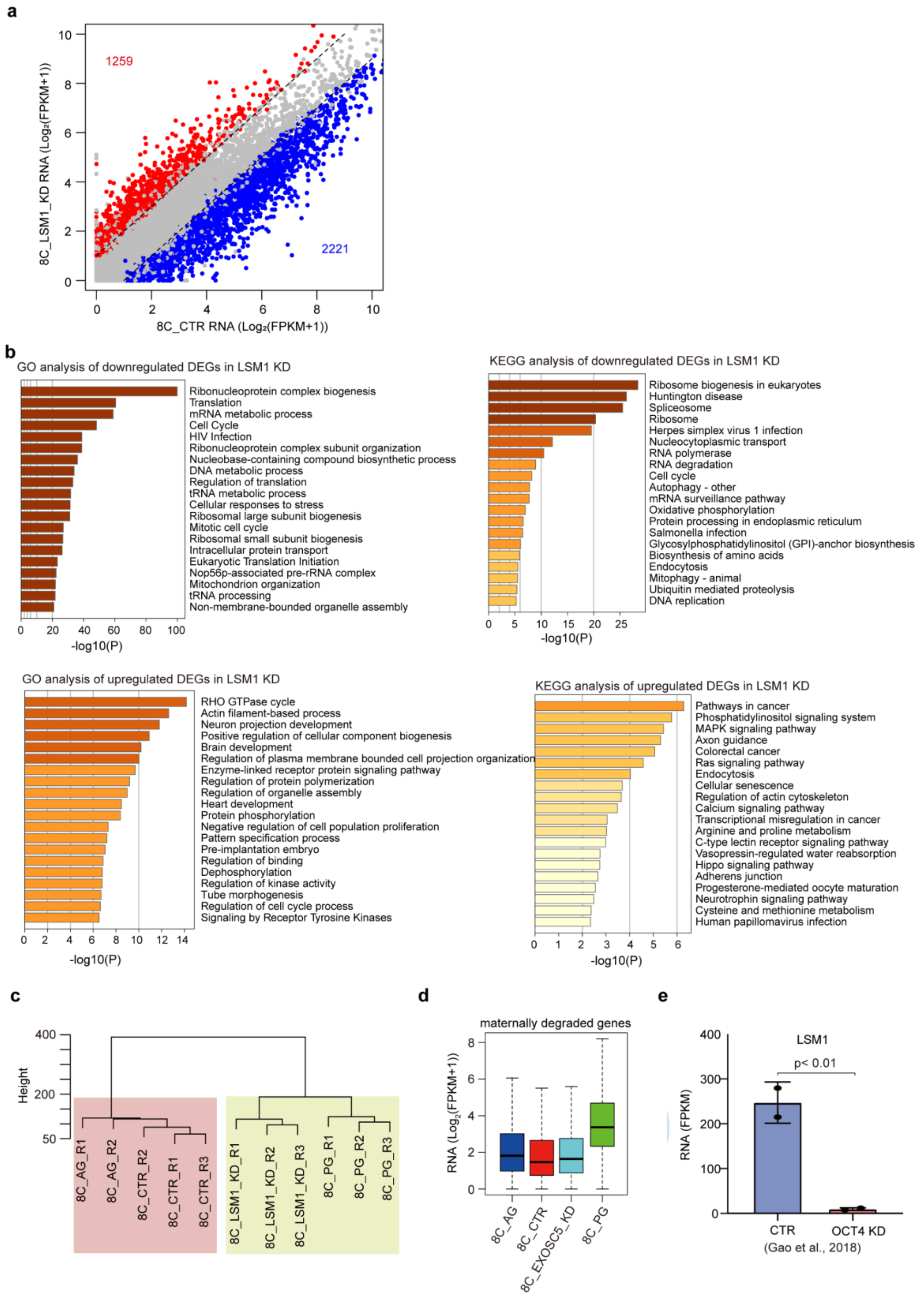

Supplementary Figure S7 LSM1 regulates ZGA and MRD in human embryo at the 8-cell stage. a Scatter plot showing the expression levels of genes between LSM1 KD and control (CTR) 8-cell embryos. The genes upregulated in LSM1 KD are labeled in red, while the genes downregulated in LSM1 KD are labeled in blue. The numbers of DEGs are indicated. **b** GO and KEGG analysis of DEGs downregulated in LSM1 KD embryos (top) and upregulated in

LSM1 KD embryos (bottom). **c** Hierarchical clustering of haploid 8-cell embryos, LSM1 KD 8-cell embryos and control bi-parental 8-cell embryos according to their RNA expression patterns. R1, R2 and R3 represent different biological replicates. **d** Boxplot comparing the expression levels of the maternal genes among AG 8-cell embryos, bi-parental 8-cell embryos, EXOSC5 KD 8-cell embryos and PG 8-cell embryos. The maternal genes refer to the genes whose transcripts are maternally deposited and degraded in bi-parental 8-cell embryos. **e** Bar plot showing the expression levels of LSM1 in control (CTR) and OCT4 KD 8-cell embryos. The RNA-seq data of OCT4 KD 8-cell embryos are obtained from (Gao et al., 2018). Error bars represent standard errors. t test was used.

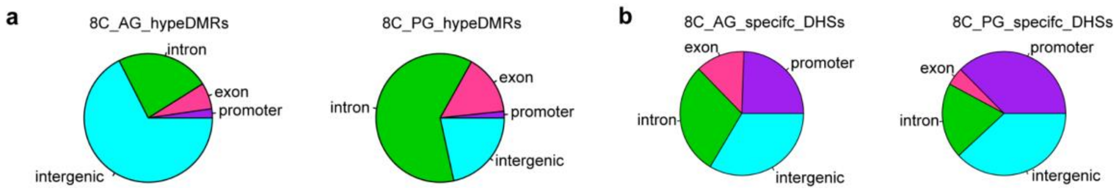

Supplementary Figure S8 The genomic distribution and of DMRs and DHSs. a The genomic distribution of AG hypermethylated DMRs (left) and PG hypermethylated DMRs (right) at 8-cell stage. **b** The genomic distribution of AG-specific DHSs (left) and PG-specific DHSs (right) at 8-cell stage.

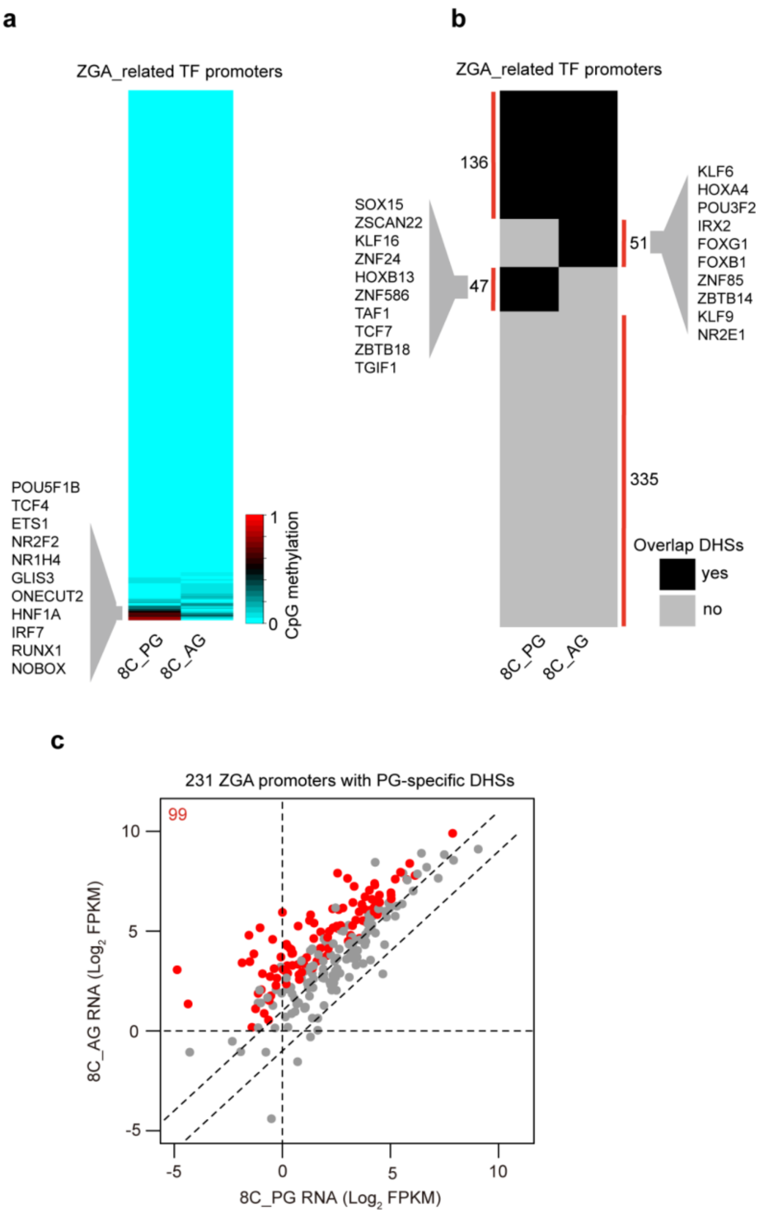

Supplementary Figure S9 Epigenetic states of ZGA-related TFs and DEGs. a Heatmap showing the DNA methylation levels (ML) of the promoters of ZGA-related TFs in PG and AG 8-cell embryos. The ZGA-related TFs with PG-specific DNA hypermethylation (ML_{PG} - ML_{AG} >= 0.3) are shown. **b** Heatmap showing the chromatin accessibility of the promoters of ZGA-related TFs in PG and AG 8-cell embryos. **c** Scatter plot showing the RNA expression levels of the ZGA genes whose promoters harbor PG-specific DHSs.

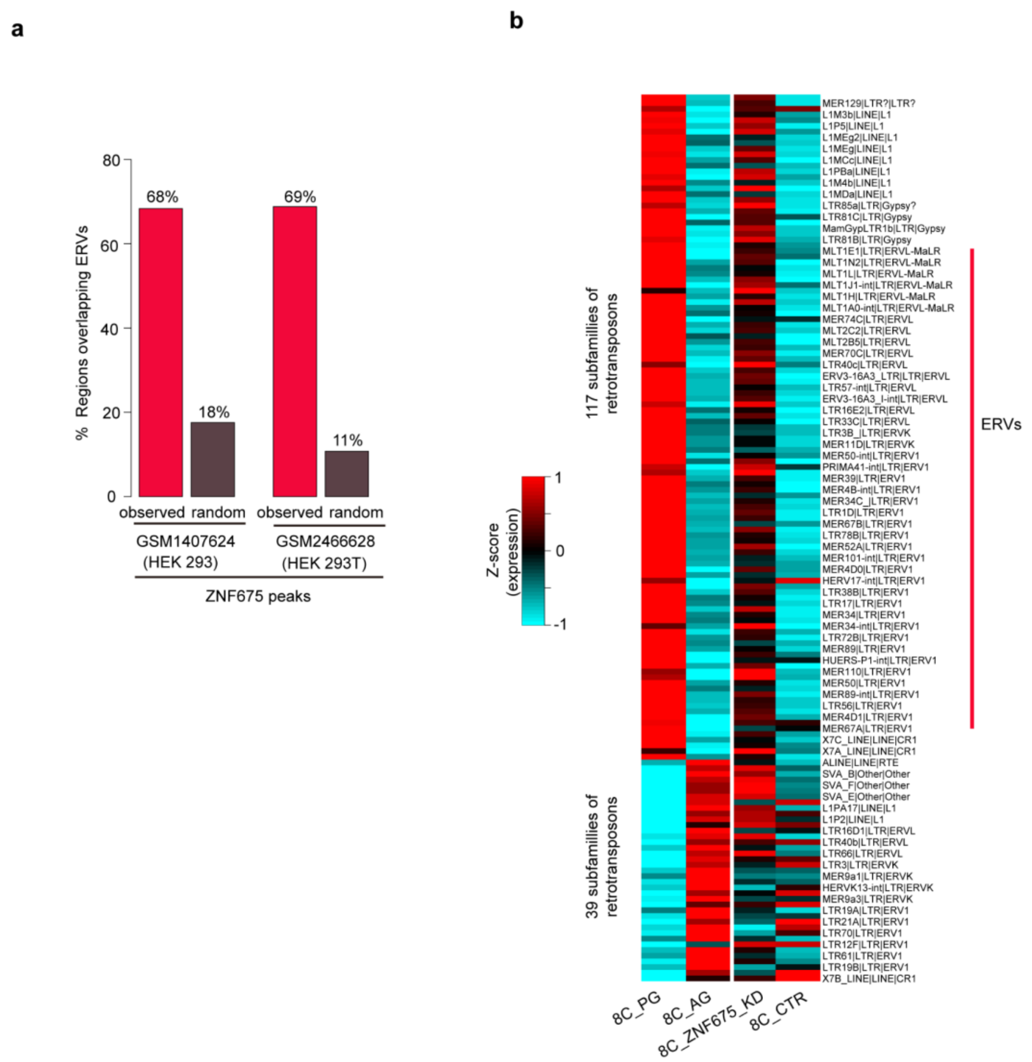

Supplementary Figure S10 The repression of retrotransposons by ZNF675 may be involved in human ZGA. a Bar plot showing the overlap between ZNF675 peaks and ERVs. The ZNF675 peaks in HEK 293 and HEK 293T cells are obtained from two previously published studies (GSM1407624, Garton et al., 2015) and (GSM2466628, Imbeault et al., 2017). **b** Heatmap showing the expression levels of retrotransposons with PG-specific expression (n=117) and AG-specific expression (n=39) at the 8-cell stages among PG and AG 8-cells embryos, as well as the ZNF675 KD and control 8-cells embryos. The expression values are scaled by z-scores. Part of the retrotransposons are labeled in the right side of heatmap.

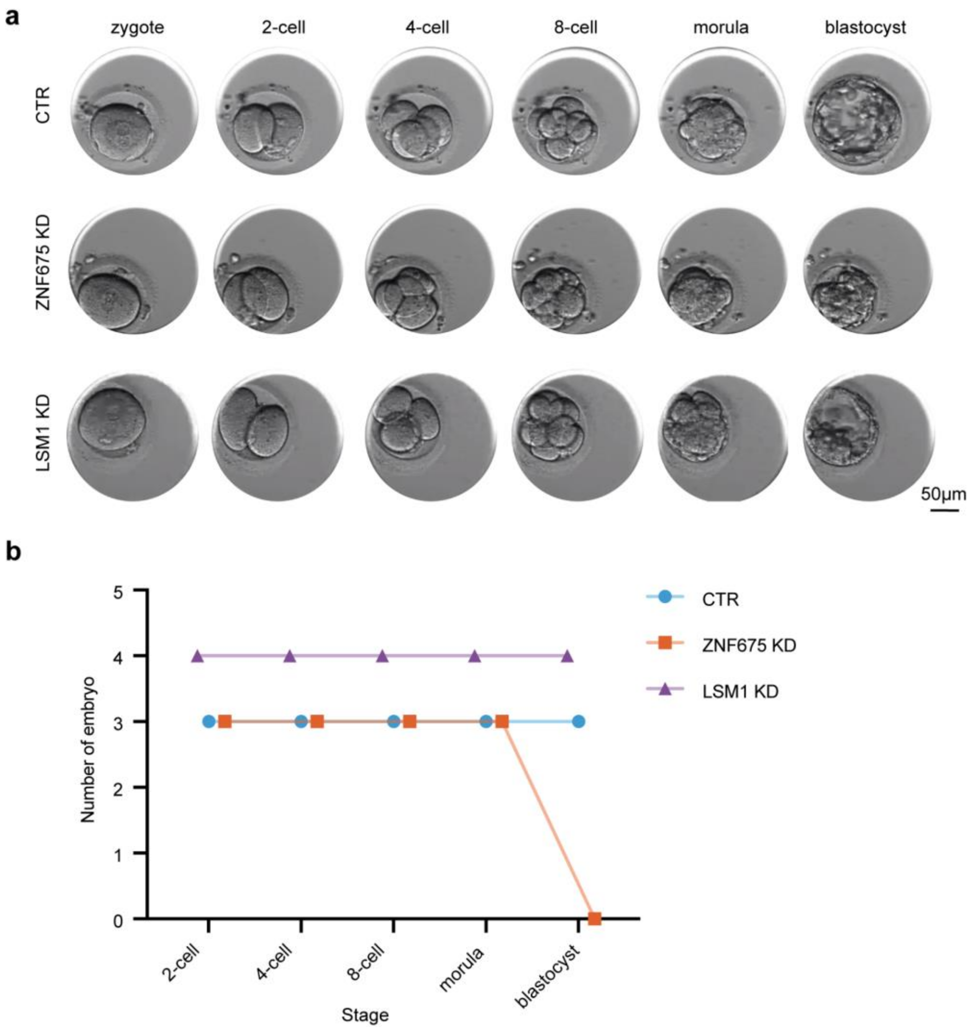

Supplementary Figure S11 The developmental potential of human embryos upon ZNF675 or LSM1 knockdown. a Representative images of control (CTR) or ZNF675 or LSM1 knockdown (KD) human embryos. Three biological replicates were performed for control or ZNF675 KD embryos. Four biological replicates were performed for LSM1 KD embryos. **b** Number of human embryos which can normally develop into the corresponding stages with normal morphological structures.
